# Inhibition of mitochondrial function by metformin increases glucose uptake, glycolysis and GDF-15 release from intestinal cells

**DOI:** 10.1038/s41598-021-81349-7

**Published:** 2021-01-28

**Authors:** Ming Yang, Tamana Darwish, Pierre Larraufie, Debra Rimmington, Irene Cimino, Deborah A. Goldspink, Benjamin Jenkins, Albert Koulman, Cheryl A. Brighton, Marcella Ma, Brian Y. H. Lam, Anthony P. Coll, Stephen O’Rahilly, Frank Reimann, Fiona M. Gribble

**Affiliations:** MRC Metabolic Diseases Unit, Addenbrooke’s Hospital, Wellcome Trust/MRC Institute of Metabolic Science (IMS), University of Cambridge, Hills Road, Cambridge, CB2 0QQ UK

**Keywords:** Cell biology, Physiology, Endocrinology, Gastroenterology, Medical research, Molecular medicine

## Abstract

Even though metformin is widely used to treat type2 diabetes, reducing glycaemia and body weight, the mechanisms of action are still elusive. Recent studies have identified the gastrointestinal tract as an important site of action. Here we used intestinal organoids to explore the effects of metformin on intestinal cell physiology. Bulk RNA-sequencing analysis identified changes in hexose metabolism pathways, particularly glycolytic genes. Metformin increased expression of *Slc2a1* (GLUT1), decreased expression of *Slc2a2* (GLUT2) and *Slc5a1* (SGLT1) whilst increasing GLUT-dependent glucose uptake and glycolytic rate as observed by live cell imaging of genetically encoded metabolite sensors and measurement of oxygen consumption and extracellular acidification rates. Metformin caused mitochondrial dysfunction and metformin’s effects on 2D-cultures were phenocopied by treatment with rotenone and antimycin-A, including upregulation of GDF15 expression, previously linked to metformin dependent weight loss. Gene expression changes elicited by metformin were replicated in 3D apical-out organoids and distal small intestines of metformin treated mice. We conclude that metformin affects glucose uptake, glycolysis and GDF-15 secretion, likely downstream of the observed mitochondrial dysfunction. This may explain the effects of metformin on intestinal glucose utilisation and food balance.

## Introduction

Metformin is the world’s most prescribed drug for type 2 diabetes mellitus (T2DM). The American Diabetes Association (ADA) and European Association for the Study of Diabetes (EASD) have recommended metformin along with lifestyle interventions for the first-line management of T2DM^1^. Metformin lowers blood glucose and insulin levels, and reduces diabetes related morbidity^2–4^. In contrast with many other antidiabetic agents, metformin also causes weight loss^5^.

Despite being used since the 1950s, the mechanisms of metformin action are still debated. Until recently it was widely accepted that the main mechanism of metformin action is by suppressing hepatic gluconeogenesis^6^. Experiments in isolated hepatocytes demonstrated that high concentrations of metformin inhibited mitochondrial oxygen consumption, through suppressing complex I of the electron transport chain^7^. Other studies have centred on the role of metformin in activating AMP-activated kinase (AMPK), either by lowering the ATP/ADP ratio or activating the upstream kinase LKB-1^8–10^. AMPK independent pathways have also been postulated, including inhibition of intracellular cAMP levels or the glycerolphosphate shuttle^11,12^. However, recent studies have reported metformin to increase, rather than decrease hepatic gluconeogenesis in T2DM patients^13,14^.

It has been argued that the gastrointestinal (GI) tract is a more important site of metformin action^15^. Measurements of ^14^C-metformin biodistribution in mice showed that within the first 4–6 h following oral gavage metformin accumulates at higher concentrations in the small intestine, compared with the liver or blood^16^. Metformin was also more effective at lowering blood glucose levels in streptozotocin induced diabetic rats when administered via an intraduodenal route compared with hepatic-portal and intravenous infusions, despite matched plasma concentrations^17,18^. Several clinical trials involving administering a “delayed-release” metformin formulation with lower bioavailability demonstrated similar efficacy as immediate release formulations in lowering blood glucose levels in patients with T2DM^19,20^.

Further implicating the intestine as an important target organ for metformin are observations from Position Emission Tomography-Computed Tomography (PET–CT) imaging in mice and T2DM patients demonstrated that oral metformin administration caused the non-metabolisable fluoro-deoxyglucose (F-2DG) tracer to accumulate in the gut, which correlates with its blood glucose lowering effect^21–25^. The weight loss but not glucose-lowering effects of metformin are attributable to increased circulating levels of Growth-differentiation factor 15 (GDF-15)^26–29^. GDF-15 is a stress-induced hormone which acts on its cognate receptor GFRAL expressed exclusively in the area postrema and Nucleus Tractus Solitarii of the brainstem to induce taste aversion^30,31^. We have previously identified the small intestine, colon and kidneys as the primary organs which elicit increased *Gdf15* mRNA expression in high fat diet (HFD)-fed mice after oral metformin gavage, suggesting that the GI tract acts as an endocrine organ in releasing GDF-15 as the mechanism of metformin-mediated weight loss^26^.

In the present study, we analysed the transcriptome and performed metabolic measurements in intestinal cultures to evaluate mechanisms of metformin action in intestinal cells, followed by confirmation of findings in 3D intestinal organoids and intestinal tissues from HFD-fed mice. Our observations suggest that mitochondrial dysfunction caused by metformin is central to its effects on glucose uptake, metabolism and GDF-15 release.

## Results

### The effects of metformin on the transcriptome of intestinal cultures

**Figure 1:**
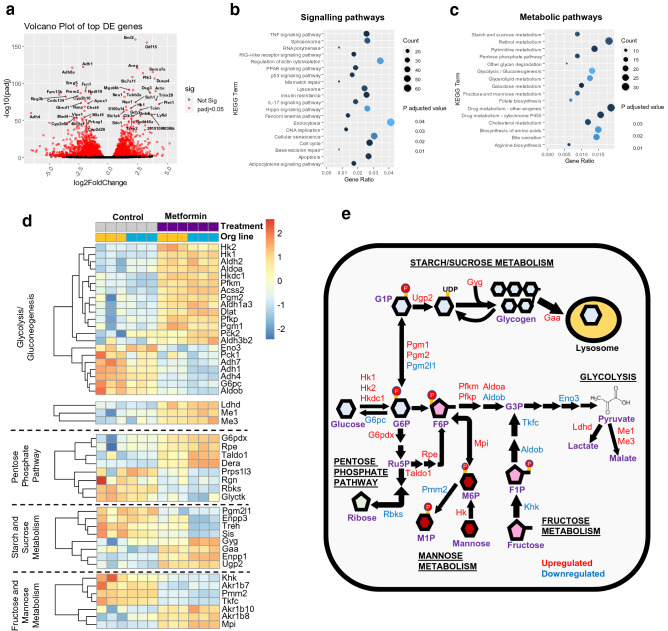
The effects of metformin on differentially expressed (DE) genes and metabolic pathways in mouse intestinal monolayer cultures. (**a**) Volcano plot displaying gene expression changes in duodenal organoid cells in 2D monolayer cultures treated with 1 mM metformin or no treatment control (n = 6 plates per condition from 2 different organoid lines). Fold-change (X-axis) compared to p-adjusted values (Y-axis) of individual genes altered by metformin treatment. Red dots represent DE genes (P < 0.05); black dots represent gene expression changes that were not statistically significant (Not Sig). Labelled genes represent the top 50 DE genes (sorted by P values). Analysis was performed using Bioconductor software packages in RStudio (v.1.2.5019), with gene annotation from the Ensembl dataset in BioMart (v2.40.5). Volcano plots were generated using the Geompoint function of the ggplot package. (**b**,**c**) KEGG enrichment analysis for changes in signalling (**b**) and metabolic (**c**) pathways affected by metformin treatment. Gene ratio is the number of DE genes in a KEGG pathway divided by total number of genes in the pathway. Count represents the numbers of DE genes. KEGG pathway analysis was performed using clusterprofiler. (**d**) Heatmaps showing DE genes (filtered by p-adjusted value < 0.05) involved in hexose metabolism. Relative expression is normalised to z-scores for each gene. Each box indicates a separate plate and the corresponding organoid line. Heatmaps were plotted using Pheatmap. (**e**) Schematic showing expression changes of genes involved in all hexose metabolism pathways. Red and blue show upregulated and downregulated genes, respectively.

To investigate the transcriptomic responses associated with metformin treatment in intestinal cells, we performed RNA-sequencing on 2D monolayer cultures of mouse duodenal organoids treated with vehicle control or 1 mM metformin for 24 h (Supplementary Figure 1). The RNA-seq analysis identified 2378 upregulated and 1660 downregulated genes associated with metformin treatment out of the 15,194 genes mapped to the database; the top 50 genes ranked based on statistical significance are depicted in a volcano plot (Fig. 1a). Using Kyoto-Encyclopedia of Genes and Genomes (KEGG)^32^ pathway enrichment analysis, we found changes in several signalling pathways, predominantly DNA or RNA repair/replication, lysosome and the cell cycle (Fig. 1b and Supplementary Figures 2–4). Amongst metabolic pathways, significant changes were observed in genes involved in hexose sugar metabolism (Fig. 1c). In glycolysis and gluconeogenesis, metformin increased the expression of hexokinase isoforms (*Hk1, Hk2* and *Hkdc1*), phosphofructokinases (*Pfkm* and *Pfkp*) and aldolase A (*Aldoa*) but decreased the expression of enolase (*Eno3*), alcohol dehydrogenases (*Adh1, Adh4, Adh7*), and the gluconeogenesis glucose-6-phosphatase enzyme (*G6pc*) (Fig. 1d,e). Metformin also altered expression of genes associated with other carbohydrate metabolic pathways (Fig. 1d,e).

### Metformin increases glycolysis in intestinal cells

**Figure 2:**
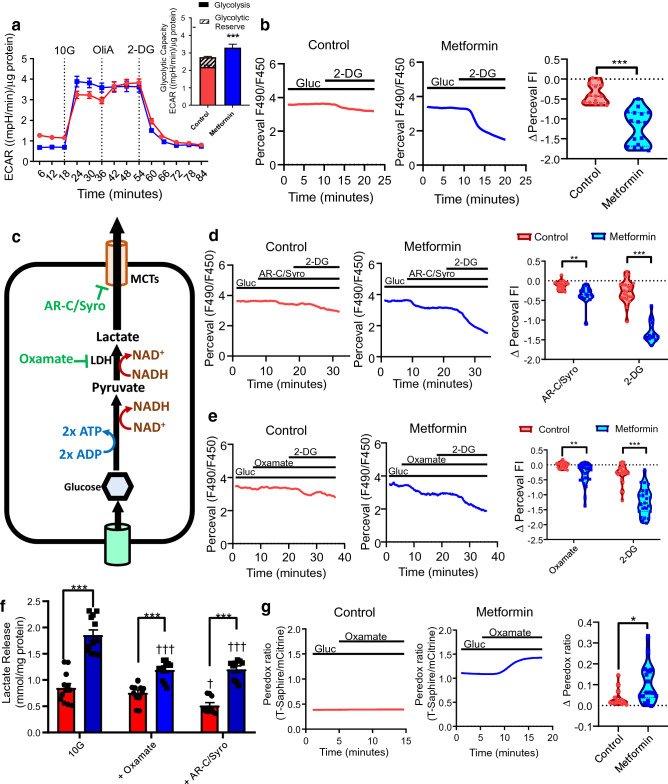
Metformin increases glycolysis in intestinal cells. (**a**) Changes in extracellular acidification rate (ECAR) during the glycolytic stress test wherein intestinal cells were pre-treated with control or 1 mM metformin for 24 h. Results were normalised to the protein concentration measured by BCA assay. Dotted lines indicate when compound were added: *Gluc* glucose (10 mM), *OliA* oligomycin-A (1 μM), *2-DG* 2-deoxyglucose (50 mM). Inset: Effect of metformin on measured parameters of the glycolysis stress test. n = 10 wells from 3 independent experiments. *NS* not significant. ***P < 0.001. Two-way ANOVA and Bonferroni post-hoc test. (**b**) Example traces of Perceval fluorescence ratios from Control and Metformin treated cells in response to 2-DG (50 mM), with violin plots shows of the change in fluorescent intensity ratio (FI). ***P < 0.001, Student’s *t* test. Control; n = 14 from 4 dishes and metformin; n = 17 from 4 dishes. (**c**) Schematic of glycolysis and effects of inhibitors. (**d**) Effects of AR-C155858 (AR-C, 10 μM) and Syrosingopine (Syro, 50 μM) on Perceval fluorescence in Control (red) and Metformin (blue) treated cells. 10 mM glucose (Gluc) was present throughout, and 2-DG (50 mM) added as indicated. Error bars are mean ± SEM. **P < 0.01, ***P < 0.001, two-way ANOVA and Bonferroni post-hoc test. Control: n = 22 cells from 6 dishes; metformin: n = 16 cells from 5 dishes. Error bars are mean ± SEM. **P < 0.01, ***P < 0.001, two-way ANOVA and Bonferroni post-hoc test. (**e**) As (**d**), for oxamate (50 mM). Control: n = 23 cells from 6 dishes; metformin: n = 25 cells from 6 dishes. (**f**) Supernatant lactate levels in control (red) and metformin (blue) pre-treated cultures in glucose (10 mM, 10G), with oxamate (50 mM) or AR-C, (10 μM) and Syro (50 μM). Error bars are mean ± SEM. *P < 0.05, **P < 0.01, ***P < 0.001, vs control. ^†††^P < 0.001 vs pre-treated conditions in 10G. Two-way ANOVA and Bonferroni post-hoc test. n = 2–3 wells from 4 independent experiments except AR–C/Syro (3 independent experiments). (**g**) Effects of oxamate on Peredox fluorescence in control (red) and metformin (blue) treated cells. Glucose (Gluc, 10 mM) and oxamate (50 mM) were applied as indicated. Error bars are median ± 95% confidence intervals. *P < 0.05, Mann–Whitney test. Control: n = 15 cells from 4 dishes; Metformin: n = 18 cells from 4 dishes.

Since metformin increased the expression of key glycolysis genes, we examined the effects of metformin on glycolysis. Metformin treated cultures exhibited increased glycolysis and reduced glycolytic reserve, as they were unable to further increase glycolytic flux as measured by ECAR upon addition of ATP-synthase inhibitor oligomycin A (Fig. 2a). Metformin modestly increased the maximum cellular capacity to perform glycolysis (Fig. 2a).

The effects of metformin on ATP generation from glucose metabolism were monitored by live-cell imaging of intestinal cells transfected with the fluorescent ATP sensor PercevalHR. The glycolysis inhibitor 2-DG decreased the Perceval fluorescence in the presence of glucose in metformin treated cells more than in control cells (Fig. 2b), confirming that metformin treated cells rely on glycolytic ATP production, whereas control cells show greater metabolic flexibility. Inhibitors of monocarboxylate transporters (MCTs) AR-C155858 (MCT1/2 inhibitor) and syrosingopine (MCT1/4 inhibitor) modestly decreased Perceval fluorescence in control cells, but the effects were more profound in metformin treated cells (Fig. 2c,d). The lactate dehydrogenase (LDH) inhibitor oxamate did not alter Perceval fluorescence in control cells, but significantly decreased Perceval fluorescence in metformin treated cells (Fig. 2c,e). Lactate release from glycolysis was increased in metformin treated cultures, whilst oxamate or AR-CC155858/syrosingopine partially reduced metformin-stimulated lactate release (Fig. 2f). In control cells, oxamate application elicited notable changes in Peredox fluorescence (a measure of the cytosolic NADH/NAD^+^ ratio) in 4/15 cells, but significantly increased Peredox fluorescence in 14/18 metformin pre-treated cells, consistent with metformin increasing cytosolic NADH generation associated with glycolysis (Fig. 2c,g).

### Mitochondrial respiration inhibitors alter the expression of glucose transporters in intestinal cultures

**Figure 3:**
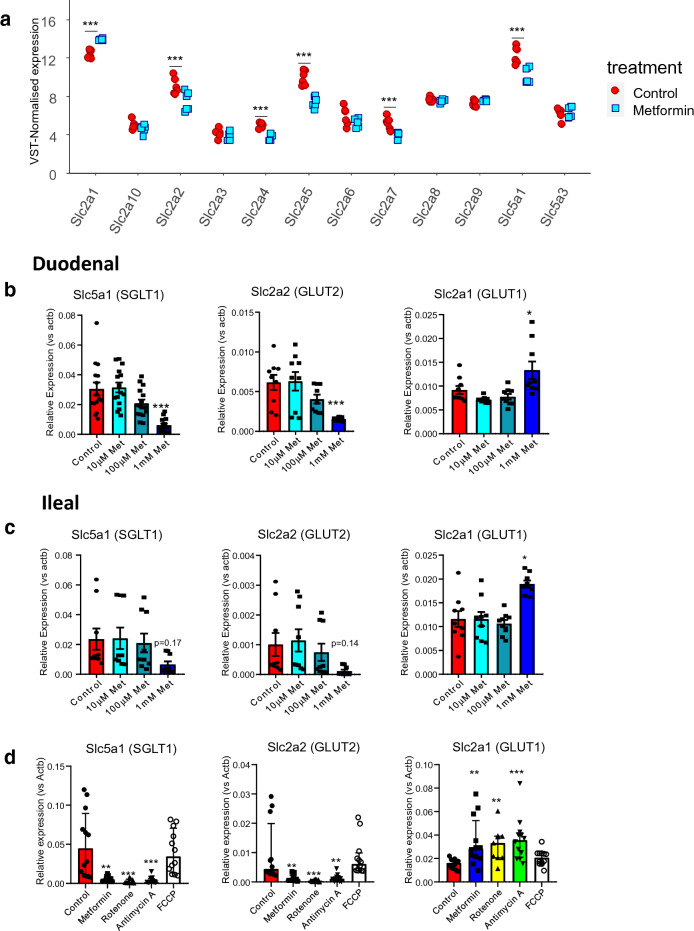
Metformin and other mitochondrial respiration inhibitors decreased expression of glucose transporters but increased Slc2a1 (GLUT1) expression. (**a**) RNA-sequencing analysis of the mRNA transcript expression of all GLUT (*Slc2aX*) transporter and SGLT (*Slc5aX*) transporter isoforms in duodenal organoid 2D monolayer cultures in response to 1 mM metformin pre-treatment for 24 h. ***P < 0.001 (p-adjusted value). DE-seq analysis and Benjamin-Hochberg corrections. Count data normalised by variance-stabilising transformation (VST) against size factors. Results are mean ± SEM. Each point represents a plate (n = 6 plates from 2 organoid lines). (**b**) qPCR analysis of glucose transporters *Slc5a1*, *Slc2a2* and *Slc2a1* in mouse duodenal organoid derived 2D monolayer cultures in response to metformin pre-treatment from 10 µM to 1 mM for 24 h. n = 9 wells from 3 independent experiments except for *Slc5a1* (n = 15 wells from 5 independent experiments). Results are mean ± SEM. *P < 0.05, **P < 0.01, ***P < 0.001. One way ANOVA and Bonferroni post-hoc test. (**c**) As (**b**) for mouse ileal cultures. n = 9 wells from 3 independent experiments. (**d**) qPCR analysis of expression of glucose transporters *Slc5a1*, *Slc2a2 and Slc2a1* in mouse duodenal organoid derived 2D monolayer cultures in response to metformin (1 mM), rotenone (10 μM), antimycin A (10 μM) and FCCP (10 μM) pre-treatment for 24 h. Results presented as median ± Interquartile range. n = 11–12 wells from 4 independent experiments. *P < 0.05, **P < 0.01, ***P < 0.001. Kruskal–Wallis and Dunn’s post-hoc test since the data did not pass normality test.

We also investigated the effects of metformin on the expression of glucose transporters, which have previously been shown to be important in regulating glycolytic flux^33^. Our RNA-seq data revealed that metformin treatment decreased the expression of GLUT family of facilitative glucose transporters *Slc2a2* (GLUT2), *Slc2a4* (GLUT4), *Slc2a5* (GLUT5), *Slc2a7* (GLUT7) and the Na^+^ coupled transporter *Slc5a1* (SGLT1) (Fig. 3a). *Slc2a1* (GLUT1), however, was upregulated by metformin treatment (Fig. 3a). The hexose transporter genes *Slc5a1*, *Slc2a1*, *Slc2a2* and *Slc2a5* were selected for further investigation since the expression levels for these genes were the highest in the RNA-seq data.

RT-qPCR analysis demonstrated that treatment with metformin at 1 mM, but not at any lower concentrations, significantly altered the expression of these glucose transporter transcripts in duodenal 2D monolayers, replicating the observations from the RNA-seq data (Fig. 3b) and similar trends were observed in ileal derived cultures, where only *Slc2a1* upregulation reached significance (Fig. 3c). 1 mM metformin also decreased the expression of the fructose transporter *Slc2a5* in duodenal and ileal organoids (Supplementary Figure 5). The mitochondrial respiration inhibitors rotenone (complex I inhibitor) and antimycin A (complex III inhibitor) but not FCCP (proton uncoupler) similarly decreased the mRNA expression of *Slc2a2* and *Slc5a1* whilst increasing the expression of *Slc2a1* (Fig. 3d). These results suggest that gene expression changes in glucose transporters after metformin treatment are associated with inhibiting the mitochondrial electron transport chain.

### Metformin increases glucose uptake mediated by GLUT transporters

**Figure 4:**
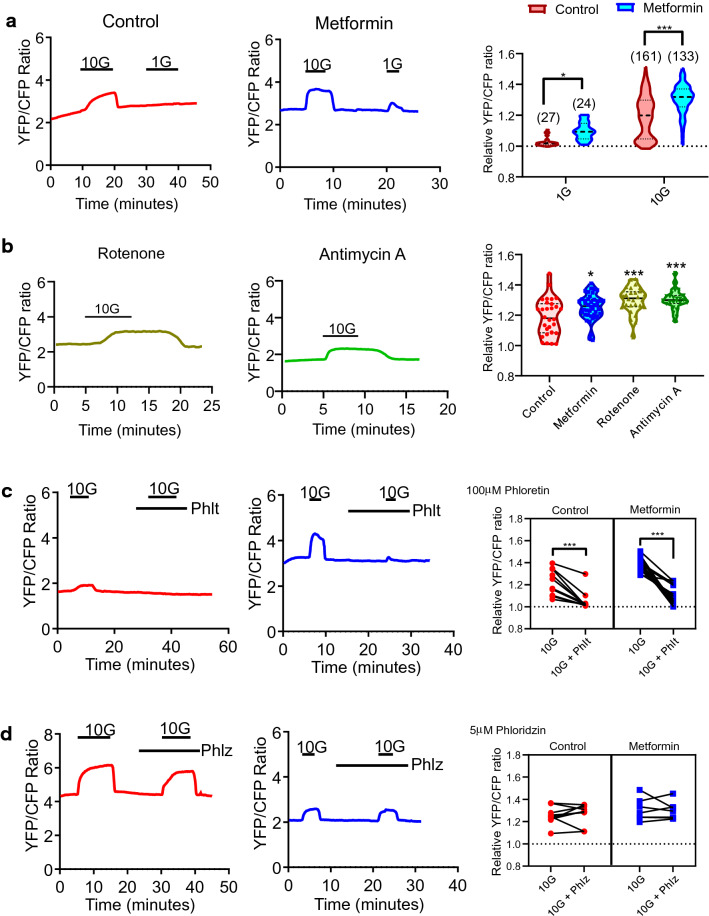
Metformin and other mitochondrial respiration inhibitors increase glucose uptake mediated by GLUT transporters in intestinal cells, measured using a FRET-based glucose sensor. (**a**) Left: Example traces of YFP/CFP fluorescence ratios from overnight control and metformin (1 mM) pre-treated cells expressing FLII12Pglu-700μδ6. Glucose was applied at 1 mM (1G) and 10 mM (10G) concentrations as indicated. Right: The effect of 1 mM and 10 mM glucose on the YFP/CFP fluorescence ratios in control (red) and metformin (blue) pre-treated cells normalised to the baseline (*P < 0.05, ***P < 0.001, Two-way ANOVA and Bonferroni post-hoc test). Results shown as a violin plot with the median and confidence intervals indicated as dotted lines. Numbers of cells studied for each condition are indicated in the graph. (**b**) Left: Example traces of YFP/CFP fluorescence ratios from overnight rotenone (10 μM) and antimycin A (10 μM) pre-treated cells. 10 mM glucose (10G) was applied as indicated. Right: The effect of 10 mM glucose on the YFP/CFP fluorescence ratios in control (red) and metformin (blue), rotenone (yellow) and antimycin A (green) treated cells normalised to the baseline (*P < 0.05, ***P < 0.001, Two-way ANOVA and Bonferroni post-hoc test). Results shown as a violin plot with the median and confidence intervals indicated as dotted lines with numbers of cells indicated in the graph. (**c**) Left: Example traces of YFP/CFP fluorescence ratios from Control and Metformin treated cells where glucose (10 mM, 10G) and phloridzin (5 µM) were applied as indicated. Right: The effect of glucose with and without 100 µM phloretin on the YFP/CFP fluorescence ratios in control (red) and metformin-treated (blue) cells normalised to the baseline. Control: n = 9 cells from 4 dishes, metformin: n = 7 cells from 3 dishes. Paired *t* test. Abbreviations: 10G; 10 mM glucose, Phlt; Phloretin. (***P < 0.001, paired-*t*-test). (**d**) Left: Example traces of YFP/CFP fluorescence ratios from Control and Metformin treated cells where glucose (10 mM, 10G) and phloridzin (5 µM) were applied as indicated. Right: The effect of glucose with and without 5 µM phloridzin on the YFP/CFP fluorescence ratios in control (red) and metformin-treated (blue) cells normalised to the baseline. Control: n = 9 cells from 4 dishes, metformin: n = 7 cells from 3 dishes. Paired *t* test. *10G* 10 mM glucose, *Phlz* Phloridzin.

The effects of metformin on glucose uptake in intestinal 2D monolayer cultures were investigated via fluorescence imaging of cells transfected with the glucose sensor, FLII12Pglu-700µδ6. Application of glucose at 10 mM increased the YFP/CFP ratio (a relative measure of increased glucose uptake) in control and metformin treated cells (Fig. 4a). Application of glucose at 1 mM concentration increased the YFP/CFP ratio in all metformin-treated, but only 4 out of 27 control cells (Fig. 4a). Metformin treatment increased glucose uptake at 1 mM and 10 mM concentrations (Fig. 4a). Similarly, rotenone and antimycin A increased glucose (10 mM) uptake by 1.3-fold (Fig. 4b).

To explore the functional identity of the glucose transporter(s) involved in metformin stimulated glucose uptake, changes in the YFP/CFP ratio were monitored in response to 10 mM glucose following pre-application of glucose transport inhibitors. Application of the broad-spectrum GLUT transport inhibitor phloretin (100 µM) reduced glucose uptake in control and metformin treated cells (Fig. 4c). This suggests that a GLUT transporter is responsible for glucose uptake. Application of the broad-spectrum SGLT inhibitor phloridzin at 5 µM, which has been reported to inhibit SGLT1 and SGLT2-mediated glucose transport in previous studies^34^, did not alter glucose uptake in either control or metformin treated cells (Fig. 4d). This suggests that the SGLT family of transporters (such as SGLT1) do not play a major role in glucose uptake in intestinal cells in the monolayer cultures.

### Metformin causes mitochondrial dysfunction in intestinal cells

**Figure 5:**
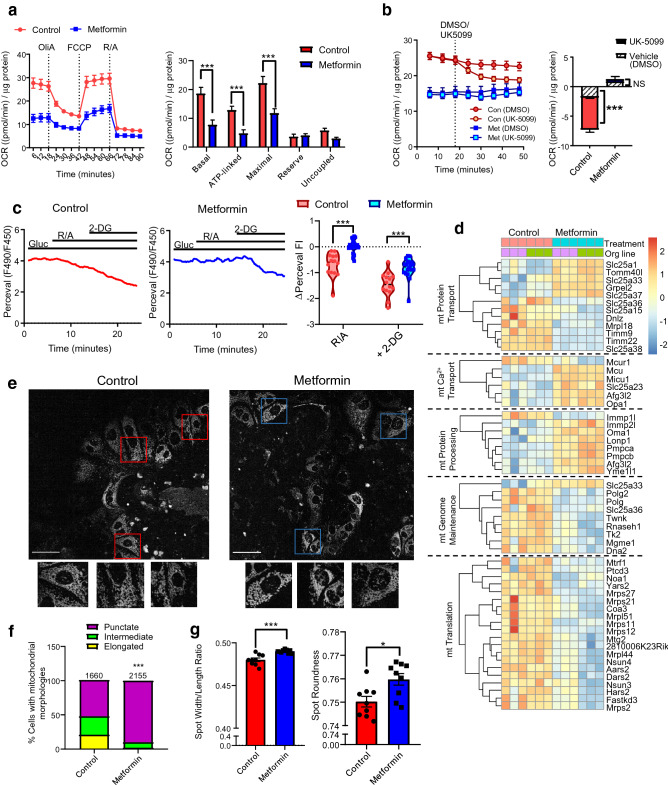
Metformin causes mitochondrial dysfunction in intestinal cells. (**a**) Left: Changes in oxygen consumption rate (OCR) during the mitochondrial stress test. Cells were pre-treated with control (ENR media) ± metformin (1 mM) for 24 h, then incubated with glucose (10 mM) in XF basal media for 1 h prior to measurements. Dotted lines indicate compound addition. Oligomycin-A (OliA, 1 μM), FCCP (1 μM), Rotenone/Antimycin A (Rot/AA, 1 μM each). Results were normalised to the protein concentration measured by BCA assay. Right: Effect of metformin on measured mitochondrial respiration parameters. n = 5 wells from 3 independent experiments. ***P < 0.001. Two-way ANOVA and Bonferroni post-hoc test. (**b**) Effects of mitochondrial pyruvate carrier inhibitor UK-5099 compared to DMSO on OCR of control and metformin overnight pre-treated cells as measured by the Seahorse bioanalyser. Results were normalised to the protein concentration measured by BCA assay. Inset: Normalised OCR was calculated as differences after drug application and third time point measured before application ***P < 0.001 compared to DMSO in control cultures. n = 4–5 wells from 4 independent experiments. (**c**) Left: Example traces of Perceval fluorescence ratios from control and metformin treated cells. Glucose (Gluc, 10 mM), Rot/AA (1 μM) and 2-DG (50 mM) were applied as indicated. Right: Effect of 10 mM glucose (Gluc), Rot/AA and 2-DG on the Perceval fluorescence ratios in control (red) and metformin (blue) overnight pre-treated cells (***P < 0.001, Repeated-measures ANOVA and Bonferroni post-hoc test. Control: n = 14 from 5 dishes, metformin: n = 20 cells from 4 dishes). (**d**) Heatmaps showing differentially expressed genes (filtered by p-adjusted value of < 0.05) involved in mitochondrial function based from the MitoCarta 2.0 dataset. Relative expression is normalised to the z-scores for each gene. Each box indicates a separate plate and the corresponding organoid line. Analysis was performed using Bioconductor software packages in RStudio (v.1.2.5019), and the heatmap generated using Pheatmap. (**e**) Confocal images of intestinal cells treated with control or metformin (1 mM) stained with MitoTracker green. Scale bar: 50 µm. Magnified images of individual cells are shown. (**f**) Quantification of the percentage of total cells with fragmented, intermediate or elongated mitochondrial in control or metformin treated cells (***P < 0.001, Chi-square test. Control: n = 1660 cells and metformin: n = 2155 cells, from 3 organoid lines). (**g**) Quantification of mitochondrial morphology as the width/length ratio or roundness by imposition of spots to image regions using the Harmonizer software.

We hypothesised that increased glucose uptake and glycolysis in intestinal cells were consequent to the established effect of metformin in inhibiting mitochondrial respiration. Mitochondrial stress test measurements showed that oxygen consumption was consistently lower in metformin treated cells in comparison to control cells in the presence of mitochondrial respiration inhibitors (Fig. 5a). Metformin treatment decreased basal, maximal, ATP-linked and uncoupled OCR, whilst the respiratory reserve was unchanged (Fig. 5a). Application of the mitochondrial pyruvate carrier inhibitor UK-5099 (10 µM) in the presence of 10 mM glucose significantly decreased OCR in control cultures, but the drug did not influence OCR in metformin treated cultures, suggesting that pyruvate generated from glucose metabolism was not mitochondrially metabolised in metformin treated cultures (Fig. 5b). Consistently, NAD(P)H and FADH autofluorescence changes in response to glucose and the mitochondrial uncoupler FCCP, which are dominated by mitochondrial redox status, showed the expected changes in control cells but were diminished in metformin treated cells (Supplementary Figure 6). Inhibition of mitochondrial pyruvate uptake using UK-5099 in the presence of glucose (10 mM) also modestly increased pyruvate levels in control cells, but not metformin treated cells (Supplementary Figure 7). The contribution of mitochondrial glucose respiration to glycolytic ATP generation was assessed by measuring Perceval HR fluorescence with rotenone and antimycin A (Rot/AA, 1 µM each). Rot/AA decreased Perceval fluorescence ratio in control cells but was without a significant effect on metformin treated cells (Fig. 5c). Subsequent exposure to 2-DG (50 mM) on top of Rot/AA markedly decreased Perceval fluorescence in both control and metformin treated cells (Fig. 5c). The impairment of mitochondrial function was not substrate specific, as the oxygen consumption rate was also significantly reduced when glutamine rather than glucose was metabolised, although the reduction in pyruvate stimulated oxygen consumption was not significantly different (Supplementary Figure 8).

We also investigated the expression profiles of genes associated with mitochondrial function in intestinal cells. Sub-analysis of expression profiles from 1158 nuclear-encoded genes associated with or localised to the mitochondria were performed from the MitoCarta 2.0 database^35^. 1056 out of 1158 genes were mapped to the database, and 289 differentially expressed (DE) genes (27.3%) were identified. GO enrichment analysis revealed upregulation of genes involved in metabolic processes, and downregulation of genes involved in RNA processes and genome maintenance (Supplementary Figure 9). Further detail of DE genes involved in mitochondrial protein import, mitochondrial calcium transport and protein processing/quality control (upregulated GO terms) and mitochondrial genome maintenance and translation (downregulated GO terms) are shown in Fig. 5d. Metformin did not elicit a notable pattern of changes in the expression of genes associated with oxidative phosphorylation (Supplementary Figure 10).

As inhibitors of mitochondrial function can cause fractured mitochondrial morphology^36^, we imaged intestinal cells stained with MitoTracker green after treatment with metformin. Control cells exhibited heterogeneous mitochondrial morphology, with 21% of cells showing predominantly elongated mitochondria, 27% intermediate and 52% punctate. By contrast, 90% of metformin treated cells had punctate mitochondria (Fig. 5e,f). Morphometric analysis of mitochondrial networks (imposed as spots to different cellular regions) showed that metformin treated cells displayed a modestly higher width to length ratio and spot roundness compared to control cells (Fig. 5g). These results demonstrate that metformin not only inhibits mitochondrial respiration in intestinal cells, but alters the expression of mitochondrial genes and mitochondrial network morphology.

### Metformin and mitochondrial respiration inhibitors induce GDF-15 in intestinal cultures

**Figure 6:**
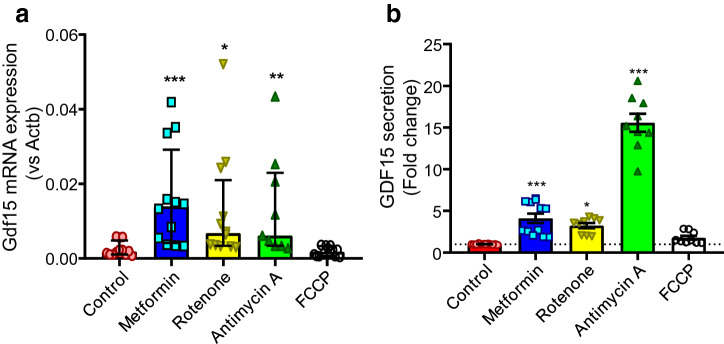
Metformin and mitochondrial electron transport chain inhibitors induce GDF-15 mRNA expression and secretion. (**a**) *Gdf15* mRNA expression in mouse duodenal 2D monolayer cultures pre-treated with no treatment control, metformin (1 mM), rotenone (10 µM), antimycin A (10 µM) and FCCP (10 µM) for 24 h (all n = 11–12 wells, 3 wells assessed in parallel from 4 independent experiments). Results presented as median ± Interquartile range. *Gdf15* expression normalised relative to *Actb*. Kruskal–Wallis test and Dunn’s post-hoc test. (**b**) GDF-15 secretion in mouse duodenal 2D monolayer cultures pre-treated with metformin (1 mM, n = 12 wells, 3 wells assessed in parallel from 4 independent experiments), rotenone (10 µM, n = 9 wells, 3 wells assessed in parallel from 3 independent experiments), antimycin A (10 µM, n = 9 wells, 3 wells from 3 independent experiments) and FCCP (10 µM, n = 9 wells, 3 wells from 3 independent experiments) for 24 h. Secretion is normalised to the control (n = 9 wells, 3 wells assessed in parallel from 3 independent experiments). Results presented as mean ± SEM. *P < 0.05, ***P < 0.001. One-way ANOVA and Bonferroni post-hoc test.

*Gdf15* was one of the most significantly upregulated genes in metformin treated intestinal 2D monolayer cultures (Fig. 1a). Consistently, 1 mM metformin elicited 4.1-fold increase in GDF-15 secretion compared to vehicle control in 2D monolayers from duodenal organoids (Fig. 6). We investigated whether in addition to metformin, other mitochondrial respiration inhibitors induce GDF-15 in intestinal cells. Like metformin, the complex I inhibitor rotenone (10 µM) induced *Gdf15* mRNA expression and elicited 3.2-fold increase in GDF-15 secretion compared to control (Fig. 6). The complex III inhibitor antimycin A (10 µM) also induced *Gdf15* mRNA expression and elicited a marked 15-fold increase in GDF-15 secretion (Fig. 6). The proton uncoupler FCCP (10 µM) neither induced GDF-15 mRNA expression or secretion. These results show that inhibitors of the electron transport chain, but not the proton uncoupler FCCP induced GDF-15 expression and secretion.

### The effects of metformin in apical-out and basal-out organoids

**Figure 7:**
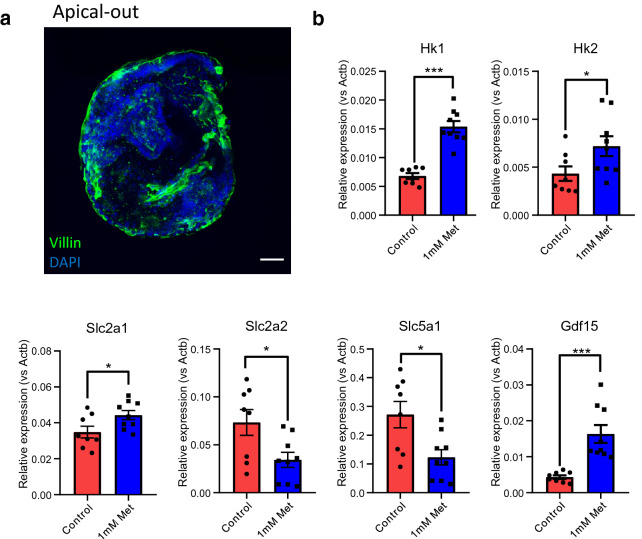
The effects of metformin on gene expression changes in apical-out and basal-out intestinal organoids. (**a**) Confocal images of mouse apical-out intestinal organoids stained with villin (green) and DAPI (blue). Scale bar = 30 µm. (**b**) qPCR analysis of glucose transporters (*Slc5a1*, *Slc2a2* and *Slc2a1*), hexokinases (*Hk1* and *Hk2*) and *Gdf15* in mouse duodenal apical-out organoids in response to metformin pre-treatment of 1 mM for 24 h. n = 8–9 wells from 3 independents. Results are mean ± SEM. *P < 0.05, **P < 0.01, ***P < 0.001. One way ANOVA and Bonferroni post-hoc test.

3D organoids have well-established apical-basolateral polarity with their apical surfaces located towards the core^37^. However, to mimic high luminal concentrations of metformin in 3D organoids, apical surfaces needs to be accessible to the media. We investigated the effects of metformin on apical-out organoids and basal-out organoids. Staining with the apical epithelial marker villin confirmed that cultured duodenal organoids in suspension for 3–4 days evert the apical membranes to the outer surface (apical-out phenotype) (Fig. 7a). After oral gavage, metformin concentrations accumulate to 0.15–3.36 mmol/kg in the small intestine in HFD fed mice^16^. We tested metformin at 1 mM as a therapeutically relevant concentration in apical-out organoids. Metformin replicated the expression changes of glucose transporters, hexokinases and *Gdf15* in apical-out organoids (Fig. 7b). We also investigated metformin at a range of concentrations in basal-out organoids. Metformin did not influence gene expression when applied at 10 µM and 100 µM concentrations (within the reported range of physiological metformin concentrations in the blood)^16^ (Supplementary Figure 11). At 1 mM, metformin significantly increased the expression of *Hk1* and *Gdf15*, but not other genes (Supplementary Figure 11). The results suggest that metformin is efficacious when exposed at a therapeutic concentration on apical-out organoids, whereas metformin applied basally (particularly at therapeutic concentrations in the blood) displayed lower efficacy.

### Oral metformin gavage replicates gene expression changes in the mouse distal small intestine

**Figure 8:**
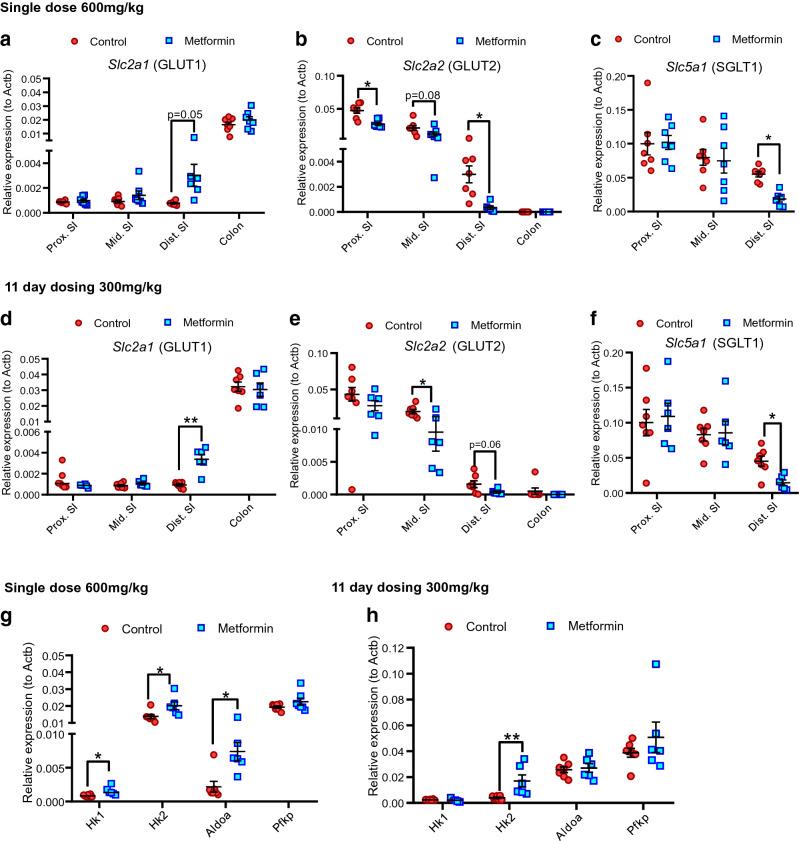
The effects of metformin on the expression of glucose transporters and metabolism genes in HFD-fed mice. (**a**–**c**) Effects of a single oral dose of metformin (600 mg/kg) on expression of glucose transporters *Slc2a1* (**a**), *Slc2a2* (**b**) and *Slc5a1* (**c**) in proximal, middle and distal small intestinal and colonic tissues of HFD-mice. mRNA analysis of fresh frozen tissue normalised to expression levels of β-actin (*Actb*). Results are mean ± SEM. n = 6 mice per group. *P < 0.05, **P < 0.01, ***P < 0.001. Student’s *t* test. *SI* small intestine. (**d**–**f**) Effects of once daily oral administration of metformin (300 mg/kg) for 11 days on expression of glucose transporters *Slc2a1* (**d**), *Slc2a2* (**e**) and *Slc5a1* (**f**) in proximal, middle and distal small intestinal and colonic tissues of HFD-mice. mRNA analysis of fresh frozen tissue normalised to expression levels of β-actin (Actb). Results are mean ± SEM. n = 6–7 mice per group (7 controls, 6 metformin). *P < 0.05, **P < 0.01. Student’s *t* test. (**g**,**h**) The effects of a single oral 600 mg/kg metformin dose (**g**) and once daily oral 300 mg/kg metformin dose for 11 days (**h**) on the expression of glucose metabolism genes *Hk1*, *Hk2*, *Aldoa and Pfkp* in the distal small intestine. Results are mean ± SEM. *P < 0.05, **P < 0.01. mRNA analysis of fresh frozen tissue normalised to expression levels of β-actin (*Actb*). Student’s *t* test.

To confirm the effects of metformin on gene expression in vivo, RT-qPCR analysis was performed on different tissue segments of intestine of HFD-fed mice orally administered metformin. 6 h after a single oral metformin dose (600 mg/kg) *Slc2a1* expression in the distal small intestine increased, but no significant changes were observed in the other regions (Fig. 8a). Metformin also decreased the expression of *Slc2a2* in the proximal and distal small intestine (with a similar trend in the middle small intestine) (Fig. 8b) whereas it decreased expression of *Slc5a1* in the distal small intestine (p = 0.05), (Fig. 8c). Once daily oral gavage of 300 mg/kg metformin for 11 days also increased the expression of *Slc2a1* in the middle small intestine (Fig. 8d). There was a trend towards decreased *Slc2a2* expression in the distal small intestine, whereas transcript expression was not affected in the proximal small intestine or the colon (Fig. 8e). Decreased *Slc5a1* mRNA expression was observed in the distal small intestine (Fig. 8f).

Since the effects of metformin on glucose transporter expression changes were predominantly observed in the distal small intestine, we investigated the effects of metformin on glucose metabolism genes in distal small intestinal tissues. A single high dose of metformin increased the expression of glycolysis genes *Hk1*, *Hk2* and *Aldoa*, but not *Pfkp* (Fig. 8g), whereas repeated lower dosing over 11 days only increased *Hk2* expression (Fig. 8h). The results demonstrated that metformin treatment for 6 h altered the expression of glucose transporter and glycolysis genes in the distal intestine in a manner similar to that observed in intestinal cultures, and that the expression profiles of glucose transporters and *Hk2* were persistent in a sub-chronic drug application regime (Fig. 8h).

Finally, we aimed to confirm previously reported preferential accumulation of metformin in the intestine compared with other tissues^16^. Five HFD-fed mice were chronically treated with metformin in the drinking water for 4 weeks and metformin concentrations in different tissues were quantified by mass spectrometry. We found relatively low plasma levels (8.5 ± 1.8 mmol/l) and concentrations only reached 0.3 ± 0.1 mmol/kg in the liver, and 0.5 ± 0.1 mmol/kg in the kidney. By contrast, substantially greater metformin accumulation was observed in the small intestine (2.8 ± 1.0 mmol/kg; 7.0 ± 3.0 mmol/kg; 12.4 ± 5.4 mmol/kg in the duodenum, jejunum and ileum, respectively) and even higher levels were found in the distal large intestine (3.9 ± 1.2 mmol/kg and 23.1 ± 2.9 mmol/kg in the proximal and distal colon, respectively).

## Discussion

In this study, we analysed the transcriptome of intestinal cultures treated with metformin. We observed that metformin: (1) increased GLUT-dependent glucose uptake, presumably via increased expression of *Slc2a1* despite decreased expression of other GLUT and SGLT family transporters, (2) increased glycolytic capacity and glycolytic ATP production and (3) increased *Gdf15* expression and GDF-15 secretion. These effects seemed to be dependent on mitochondrial dysfunction, since other electron transport chain inhibitors replicated the effects of metformin on gene expression, and metformin decreased glucose oxidation, altered the transcription of mitochondrial genes, and fractured mitochondrial networks. We replicated the effects of metformin on expression changes of glucose transporters, hexokinases and *Gdf15* in 3D intestinal organoids and the distal small intestinal tissue of HFD-fed mice.

Previous transcriptomic profiling to investigate mechanisms of metformin action have been performed on hepatocytes, skeletal muscle and adipose tissue biopsies, but not intestinal cells^38,39^. However, observations that metformin accumulates at the highest levels in the GI tract after oral administration, replicated here after chronic dosing in mice, and clinical studies involving “delayed release” formulations of metformin argue that the GI tract plays an important role in mediating the pharmacological effects of metformin^16,19,20,40^. Amongst the prominent findings of the effects of metformin on intestinal cells were alterations in hexose metabolism pathways, notably upregulation of key genes involved in glycolysis.

Previous studies investigating the effects of metformin on glucose handling in the small intestine have postulated both altered intestinal glucose metabolism as well as basal-to-apical export of glucose into the lumen via AMPK-dependent GLUT2 expression on the brush-border membrane^41–44^. Our metabolic measurements showed that metformin increased intestinal glycolysis by increasing glycolytic capacity, increasing the dependence of intestinal cells to produce ATP from glycolysis and lactate release. Recent studies involving metabolomics measurements of arterial-venous differences in pigs revealed that visceral organs (including the GI tract) are the biggest consumers of glucose (60%) and amino acids^45^. Furthermore, Schommers et al*.*, used metabolic tracing in HFD-fed mice to demonstrate that metformin increases glucose metabolism to lactate in the intestine as a result of inhibiting mitochondrial function, resulting in a futile cycle as lactate is reconverted to glucose by the liver^43^, and potentially contributing to increased energy expenditure. These studies provide in vivo evidence supporting our observations in intestinal 2D cultures to suggest the importance of intestinal glucose metabolism in metformin action.

In addition to glycolysis, we observed that metformin increased the expression of *Slc2a1* (GLUT1) and increased GLUT-dependent glucose uptake in 2D monolayers, 3D organoids and the distal small intestine of metformin treated mice. It is tempting to speculate that an increased intestinal glucose utilization plays an important part in the blood glucose lowering action of the drug^21–25,46^. Studies by Penicaud et al*.*, showed that intestinal glucose utilisation correlated with the hypoglycaemic effects of metformin when given to female obese rats for 8 days^46^. PET–CT imaging studies showed that long-term oral metformin administration increased accumulation of the intravenously injected non-metabolisable glucose analogue 18fluoro-deoxyglucose (^18^F-DG) in the small intestine and colon^21–25^. A recent phase 2 clinical trial in T2DM patients reported that under euglycaemic hyperinsulinaemic conditions, metformin increased ^18^F-DG uptake in the small intestine and colon in an insulin independent manner, and colonic glucose uptake was positively correlated with reduced fasting plasma levels in the metformin cohorts^24^. Our observations suggest a molecular explanation for the gastrointestinal tract as a “sink” in depositing glucose from the circulation as a mechanism of achieving glucose-lowering effects of metformin^24^.

The observations that metformin decreased the expression of glucose absorption transporters *Slc2a2* (GLUT2) and *Slc5a1* (SGLT1) could potentially suggest that metformin decreases transepithelial glucose transport. Previous studies in mice and T2DM patients demonstrated that another mechanism of metformin action was to decrease intestinal absorption of ingested glucose and increase intestinal glucose transit^47,48^. Yet, our findings do not support other reports showing that metformin increased GLUT2 abundance in the intestinal epithelium^42,44^.

In addition to inhibiting glucose oxidation as expected from the well-established effects of metformin on complex I inhibition^49–51^, we showed that metformin caused transcriptomic changes in mitochondrial genes and punctate mitochondrial morphology. The importance of complex I inhibition in the anticancer actions of metformin have recently been discussed^52^. In addition, RNA-sequencing data of mitochondrial gene sets showed that metformin increased the expression of genes involved in mitochondrial calcium processes and mitochondrial protein quality control, whilst decreasing the expression of genes involved in mitochondrial DNA replication and translation. A recent study by Loubiere et al., demonstrated that metformin stimulated mitochondrial Ca^2+^ uptake in LNCaP prostate cancer cells, which promotes mitochondrial swelling and disorganisation of cristae, suggesting a role of mitochondrial Ca^2+^ signalling in mitochondrial stress or dysfunction in intestinal cells^36^. Insights from mouse models harbouring mutations in genes involved in DNA replication or translation revealed punctate mitochondrial morphology, as well as altered mitochondrial one-carbon pool and increased expression of mitochondrial quality control genes such as *Lonp1*, *Yme1l1* and *Oma1*^45,53,54^. We also observed upregulation of lysosome-related genes, which could play a role in mitochondrial fragmentation^55^. Therefore, the dysregulation of other mitochondrial processes by metformin could contribute to fractured mitochondrial morphology.

We and others have previously established the importance of GDF-15 in mediating the effects of metformin on food intake and weight loss. Our observations showed that the small intestine and colon, as opposed to the liver, played an important role in mediating the effects of metformin in increasing GDF-15 through the activation of the integrated stress response (ISR)^26,29^. Here we identified *Gdf15* as one of the most significantly upregulated metformin responsive genes. This corroborated transcriptomic data from primary hepatocytes, which revealed that metformin increased *Gdf15* expression independent of the AMPK signalling pathway^39^. Our results provide further evidence that metformin-elicited GDF-15 induction was dependent on mitochondrial dysfunction, as other electron transport chain inhibitors rotenone and antimycin A also induced GDF-15 mRNA and secretion. FCCP did not increase GDF-15 secretion, suggesting that the mechanism involved inhibition of the electron transport chain or oxygen consumption as opposed to depletion of ATP levels. These findings corroborate other observations that *Gdf15* mRNA expression was upregulated in mouse models and patients with mitochondrial diseases^54,56,57^. We found that rotenone and antimycin A also altered the expression of glucose transporters and increased glucose uptake to a similar extent as metformin. This could explain the observations that metformin application at 1 mM (concentrations sufficient to inhibit mitochondrial function), but not at any lower concentrations, altered the expression of glucose transporters^7,49–51^. Our observations suggest that mechanisms of metformin in increasing glucose uptake and GDF-15 are both dependent on mitochondrial dysfunction.

Previous studies in Caco-2 cells seeded in transwells demonstrated that uptake of metformin was most efficient when applied to the apical as opposed to the basolateral surface, which could partially be explained by higher apical expression of OCT1, PMAT and SERT^58,59^. Our observations demonstrated that apical-out organoids replicated the effects of metformin on the mRNA expression of glucose transporters, hexokinases and *Gdf15* in 2D monolayer cultures. We also found that metformin increased *Gdf15* and *Hk1* in basal-out organoids at the same concentrations, which could suggest some metformin uptake in the basolateral membrane of organoids, or that the leaky nature of intestinal organoids could enable metformin to reach the apical membrane^60^. However, the observations that metformin application at 10 µM and 100 µM were without effect on gene expression in basal-out organoids might suggest that the previously reported therapeutic metformin concentrations in the blood would be insufficient to elicit changes in gene expression^16^; it is tempting to speculate that the exposure of intestinal epithelial cells to the much higher luminal metformin concentrations is critical for the therapeutic effect.

We also reported that the expression of glucose transporters and hexokinase gene *Hk2* were replicated in the distal small intestine of HFD-fed mice treated with oral gavage for 6 h and 11 days. Our previous study using the same mice also demonstrated that metformin increased *Gdf15* expression in the proximal and distal small intestine and colon^26^. It should be noted that metformin only altered the expression of the glucose transporters (especially *Slc5a1* and *Slc2a1*) in the distal small intestine, whereas the effects in other intestinal regions were highly variable. Biodistribution studies by Wilcock and Bailey demonstrated that metformin accumulates at the highest concentrations in the distal small intestine compared to other intestinal regions after metformin oral gavage in mice^16^. These regional differences are likely to be explained by variations of metformin biodistribution in the GI tract as opposed to differences in metformin action in different regions of the intestinal tract, since the expression of glucose transporters was similar in duodenal and ileal 2D monolayers treated with different concentrations of metformin.

In summary, through transcriptomic analysis and functional studies in intestinal cells, we demonstrate that mitochondrial dysfunction triggered by high concentrations of metformin exposure was associated with GLUT1-dependent glucose uptake, glycolysis and GDF-15 release. These cellular mechanisms could explain the direct roles of metformin in mediating intestinal glucose utilisation and weight loss.

## Materials and methods

### Intestinal organoid and 2D monolayer cultures

Generation and maintenance of mouse intestinal organoids were performed as previously described^61,62^. Duodenal and ileal organoids were generated from isolated intestines from the proximal 3 cm beyond the stomach and distal 10 cm to the ileal-caecal junction, respectively. Organoids were suspended in domes containing Basement Material Extract (BME, Cultrex PathClear Reduced Growth Factor Type 2, R&D Systems) with growth media containing Epithelial Growth Factor (EGF), Noggin, R-Spondin (= ENR) and Rho-associated protein kinase (ROCK) inhibitor Y-27632 (10 µM). 2D monolayer cultures were established as previously described^61^. Briefly, organoids were dissociated using TryPLE reagent (Life Technologies) for 2–4 min at 37 °C followed by mechanical trituration, before seeding the suspension on dishes coated with 2% matrigel dissolved in Advanced DMEM/F12 (4500 mg/l glucose, Life Technologies) media.

### Bulk RNA-sequencing

In preparation of samples for RNA-sequencing, two transgenic organoid lines (called GIP-cre tdRFP and SST-cre tdRFP), which express cell specific fluorescent reporters in enteroendocrine cell populations but are otherwise normal, were plated as 2D monolayer cultures in 3 separate 24-well plates on day 1 followed by treatment with or without 1 mM metformin for 24 h. Mouse intestinal 2D monolayer cultures plated were lysed in RLT plus buffer and the RNA was extracted using the RNeasy Micro Plus kit (Qiagen) according to manufacturer’s instructions. Removal of salt carryover was subsequently performed using RNeasy MinElute Cleanup kit (Qiagen). The quality of RNA was validated by Bioanalyser RNA Nano kit (Agilent) and Agilent Bioanalyser 2100 system with RIN values between 8.1 and 9.4. 1 µg of total RNA was used for library construction using Illumina's TruSeq Stranded mRNA Library Prep Kit according to the manufacturer’s protocol at the Institute of Metabolic Science Genomics and Transcriptomics Core Facility (Cambridge, UK). Indexed libraries were purified, normalised, pooled and sequenced on the HiSeq 4000 platform (Illumina) at the Genomics Core Facility, Cancer Research UK Cambridge Institute (Cambridge, UK).

All RNA sequencing analyses were performed using Bioconductor software packages in RStudio (v.1.2.5019) Gene annotation was obtained from the Ensembl dataset held in BioMart (v2.40.5). Differential expression of genes was calculated using the DESeq2 package (v1.24.0). A first DESeq analysis was performed to obtain a list of non-differentially expressed (non-DE) genes (P adjusted value > 0.05) between control and metformin treated samples pooled from both organoid lines combined by fitting a negative binomial generalised linear model. A second DESeq analysis was performed by estimating the size factors using only the non-DE genes from the first analysis. A local dispersion estimation fit was performed in the second DESeq analysis. A threshold with log2FoldChange of 0.3 was selected and any genes with < 0.3 log2FoldChange were classified as non-DE genes. The raw counts were normalised by variance stabilising transformation (VST), which divides the raw count data by the Size Factors. Gene Ontology and KEGG pathway analysis were performed using goseq and clusterprofiler packages, respectively. Mitochondrial gene lists were generated from the MitoCarta2.0 database^35^.

### Real-time quantitative PCR (RT-qPCR)

RNA extraction from mouse intestinal 2D monolayer cultures plated in 48 well plates was performed using the RNeasy Micro Plus kit (Qiagen) according to manufacturer’s instructions. RNA-extraction from frozen intestinal tissues of HFD-fed mice was performed as described^26^. The reverse transcription reaction was performed using MMLT-RV transcriptase (Promega) on the Peltier-Thermo Cycler-225 (MJ Research) according to manufacturer’s instructions. RT-qPCR reactions were performed as described^63^. The Taqman probes (Thermo Fisher) were Actb (Mm02619580_g1), Slc2a1 (Mm00441480_m1), Slc2a2 (Mm00446229_m1), Slc5a1 (Mm00451203_m1), Slc2a5 (Mm00600311_m1), Gdf15 (Mm00442228_m1), Hk1 (Mm00439344_m1), Hk2 (Mm00443385_m1), Pfkp (Mm00444792_m1), Aldoa (Mm00833172_m1). The RT-qPCR reaction was performed using the 7900 HT Fast Real-Time PCR system (Applied Biosystems, Fisher Scientific, Waltham, MA, USA). The qPCR results were normalised by calculating the difference in cycle threshold values (ΔCT) between the housekeeper gene β-actin and the gene of interest (CT_βactin_ – CT_gene_). Relative gene expression was expressed as 2^ΔCT^ for the given gene.

### Transfection of 2D monolayer cultures

For live-cell imaging studies, on day 2 after establishing 2D monolayer cultures and before drug pre-treatment, cultures were transfected with the plasmid of interest. A transfection mix was prepared by mixing 2 µg of plasmid DNA with 2 µL of Lipofectamine 2000 reagent (Invitrogen) in 100 µL of Opti-Mem (Thermo Fisher Scientific) per reaction and incubated for 10 min at room temperature. 100 µL of transfection mix was added dropwise onto the centre of the imaging dish and incubated at 37 °C for 4–8 h. Transfection efficiency was visualised using the EVOS cell imaging system (Thermo Fisher Scientific). Transfected cells were overnight (18–28 h) treated with or without metformin/mitochondrial respiration inhibitors before imaging.

### Live cell imaging of glucose and pyruvate levels

Live-cell imaging of glucose uptake was performed in cells transfected with FLII12Pglu-700μδ6 FRET-based glucose sensor, and live-cell imaging of pyruvate levels was performed in cells expressing the FRET-based sensor Pyronic^64,65^. Imaging of fluorescent-resonance energy transfer (FRET) sensors was performed on 2D monolayer cultures 2 days after seeding in 35 mm glass bottomed dishes and a day following transfection of the FRET sensor. Imaging was performed using an inverted fluorescence microscope (Olympus IX71) with a 40 × oil-immersion objective lens. Cells were excited at 435 ± 10 nm using a 75 W xenon arc lamp connected to a monochromator (Cairn Research), controlled by the MetaFluor software (Molecular Devices). CFP or mTFP emissions at ~ 470 nm and YFP or Venus emissions at ~ 535 nm were simultaneously monitored using an Optosplit II beam splitter (Cairn Research) and Orca ER camera (Hamamatsu Photonics). Images were acquired every 5 s.

### Live cell metabolic imaging of Perceval HR and Peredox sensors

Perceval HR is a genetically encoded fluorescent intracellular ATP and ADP sensor as described in^66^. Transfected cells expressing Perceval HR (21737, Addgene) were imaged at 490 ± 2 nm and 450 nm excitation sequentially, and images were acquired every 10 s. Images were background subtracted using MetaFluor software. Fluorescence emitted from excitation at 490 nm was dictated by changes in intracellular ATP concentrations, whilst the isosbestic point remained constant. The Perceval fluorescence ratio (FI/F0) was calculated by dividing the fluorescence emitted from excitation at 490 nm by 450 nm.

Peredox is a genetically encoded fluorescent sensor exclusively localised in the cytosol and not in the mitochondria, enabling compartmentalised imaging of cytosolic NADH concentrations (as a measurement of the cytosolic NADH/NAD^+^ redox ratio)^67^. The T-Sapphire and mCitrine fluorescence of the Peredox sensor (32386, Addgene) was sequentially excited at 405 ± 20 nm and 480 ± 10 nm respectively, and images were acquired every 10 s. T-Sapphire fluorescence (emitted from excitation at 405 ± 20 nm) was increased correlating with cytosolic NADH concentrations, whilst the mCitrine fluorescence remained constant throughout the experiment. Peredox fluorescence ratio (FI/F0) was calculated by dividing the fluorescence at 405 nm by 480 nm.

### Autofluorescence imaging of NAD(P)H and FAD

The cellular redox states can be estimated by measuring NAD(P)H and FAD autofluorescence^68^. NAD(P)H has an autofluorescence characteristic of the nicotinamide ring at UV range of excitation, whilst FAD has a characteristic autofluorescence of the flavin ring excited at ~ 450 nm. Autofluorescence imaging was performed on untransfected 2D monolayer cultures 2 days after seeding in 35 mm^2^ glass bottomed dishes. A phase contrast image of the cells was captured as a reference to identify cells/regions of interest. NAD(P)H and FAD autofluorescence were sequentially excited at 360 ± 15 nm and 465 ± 10 nm, respectively and images were acquired every 10 s. Background subtraction was performed using MetaFluor software and fluorescence measurements were transcribed in an excel spreadsheet. Data points were smoothened with a sliding average of 60 s. Changes in NAD(P)H and FAD autofluorescence (measured as arbitrary units) were calculated by subtracting the differences from the maximal fluorescence response during treatment to the maximal fluorescence 120 s prior to treatment (basal).

### Seahorse bioanalyser assays

Experiments were performed on the Seahorse XF24 bioanalyser (Agilent) according to manufacturer’s instructions. Briefly, 20 of the 24 wells in the XF24 plates (Agilent) were used to establish 2D monolayer cultures at full confluency, with 4 wells used as blanks (no cell controls). The cultures were treated with/without metformin in a random plate order for 24 h at 37 °C in the humidifying chamber in 5% CO_2_. On the day of the experiment, the cells were incubated with 525 µL of Seahorse XF base medium without Phenol red (Agilent) with the indicated compounds for 1 h, 37 °C in a non-CO_2_ incubator. The cartridge was loaded with 75 µL of the indicated drugs. All experiments were performed at 37 °C. At each measurement of extracellular acidification rate (ECAR) and oxygen consumption (OCR), each well was mixed for 3 min followed by measurements for another 3 min. ECAR and OCR were normalised to total protein content by performing BCA assay (Sigma) on lysed cells at the end of each experiment.

### MitoTracker imaging and mitochondrial morphology analysis

Intestinal cells were seeded in 2D monolayers in black walled 96 well plates (Perkin Elmer) prior to treatment with or without 1 mM metformin for 24 h. Cells were stained with MitoTracker green (Thermo Fisher) for 20 min, followed by incubation with ENR media for another 30 min. Cultures were washed at least 3 times with media for 2 h (where 1 mM metformin was present throughout each wash step in treatment groups). Live cell confocal imaging was performed on a high-content imaging platform at × 40 objective (Opera Phenix-Perkin Elmer). Mitochondrial morphology analysis was performed using Harmony software. Images were background subtracted using Sliding Parabola function, followed by defining regions with mitochondrial networks based on images taken at DIC, as well as criteria including surface texture, brightness and sizes of regions and linear classifier machine learning. Spots were applied onto mitochondrial networks, and geometrical parameters were calculated based on the shapes of spots. Separately, cells were also categorised and counted (using Fiji) by a condition-blinded experimenter scoring mitochondrial morphological characteristics: elongated (where all of the mitochondria displayed strand like patterns), intermediate (where mitochondria displayed a mixture of fragmented and elongated features) and fragmented (where mitochondria displayed dot-like patterns).

### Lactate release assays

Lactate release was performed 2 days after seeding mouse intestinal organoids into 2D monolayers in 48 well plates and 24 h after treatment with/without 1 mM metformin. On the day of experiment, media was removed and cultures were washed 3 times with warm PBS. Cultures were incubated for 4 h at 37 °C in the humidifying chamber treated with 100 µL of DMEM without phenol red, glucose, glutamine or pyruvate (A1443001, Thermo Fisher) plus the indicated drugs. The medium was then collected and spun at 5000*g* for 5 min at 4 °C to remove debris and dead cells. The supernatant was collected and lactate was measured at the Core Biochemical Assay Laboratory, University of Cambridge using a lactate assay kit (Siemens Healthcare). The lysates were treated with lysis buffer, and lysates were collected and spun at 10,000*g* for 10 min at 4 °C. Secretion results were normalised to total protein lysate content measured using BCA assay (Thermo Fisher).

### GDF-15 secretion assays

GDF-15 secretion was performed a day after seeding mouse intestinal organoids into 2D monolayers in 48 well plates. 2D organoid cultures were treated with mitochondrial respiration inhibitors for 24 h at 37 °C in the humidifying chamber with 150 µL of ENR and the indicated drugs. The medium was then collected and spun at 5000*g* for 5 min at 4 °C. The supernatant was collected and GDF-15 was measured at the Core Biochemical Assay Laboratory, University of Cambridge using a mouse GDF-15 assay kit (MGD-150, R&D Systems) using a microtiter plate-based two-site electrochemiluminescence immunoassay (MesoScale Discovery assay). The secretion results were normalised to basal secretion (control) for each experiment to calculate the fold change.

### Generation of apical-out and basal-out organoids

The protocol for generation of apical-out organoids was adapted from^37^. Briefly, 24 well plates were coated with 1% PolyHEMA (Sigma) dissolved in absolute ethanol and left to dry overnight to produce low attachment plates. Organoids were collected in 5% EDTA dissolved in PBS or Cell Recovery solution (Corning) in lo-bind Eppendorf tubes and placed on a plate rotator for 1 h at 4 °C to remove BME. The organoids were then spun at 600 × *g* for 5 min and the pellet was resuspended in ENR before plating in low-attachment plates. The organoids were cultured for 2–3 days prior to treatment with 1 mM metformin for 24 h before RNA extraction. Basal-out organoids were cultured by suspending organoids in BME and plated as domes in 48-well plates (as described in “Metformin increases glycolysis in intestinal cells”).

### Immunohistochemistry and confocal imaging

Apical-out organoids were collected, spun at 500 × *g* for 5 min and washed with PBS, whilst basal-out organoids were collected in Cell recovery solution for 30 min on ice to remove BME before spinning. Organoids were fixed with 4% Paraformaldehyde (Alfa Aesar) at room temperature for 30 min. After 3 × 10 min wash (PBS and 0.1% Triton X-100, Sigma), organoids were blocked with donkey serum (Sigma) for 1 h at room temperature followed by staining with Rabbit anti-villin antibody (1:1000, Abcam) at 4 °C overnight. The next day, organoids were brought to room temperature for 1 h, 3 × 10 min PBS and 0.1% Triton X-100 wash, followed by application of anti-rabbit Alexa-Fluor 488 secondary antibody (1:300, Invitrogen) for 1 h at room temperature. Organoids were washed in PBS and 0.1% Triton X-100 and treated with 2 µg/mL DAPI (1:300, Sigma) before mounting in Hydromount (National Diagnostics). Organoids were imaged using Leica SP8 laser-scanning confocal microscope (Leica) and z-stack images were taken with 1 μM thickness. Images were analysed via Fiji.

### High fat diet mouse experiments

The experimental procedures used in experiments involving male C57BL6/J mice fed with HFD were described previously^26^. All mouse studies were performed in accordance with UK Home Office Legislation regulated under the Animals (Scientific Procedures) Act 1986 Amendment, Regulations 2012, following ethical review by the University of Cambridge Animal Welfare and Ethical Review Body (AWERB). In brief, samples were taken from two cohorts; one cohort where mice had been fed on HFD for 4 weeks (1 week 45% HFD, 3 weeks 60% HFD) and given a single dose of 600 mg/kg with tissue samples taken 6 h later, the second cohort where mice switched to 60% HFD for 3 days before given 300 mg/kg does for 11 days with tissue taken 4 h after last dose.

Intestinal tissue was isolated from mice as previously described in^26^. The small intestine (from the stomach to the ileal-caecal junction) was isolated into three equal length segments and intestinal tissues were taken from the middle of each segment. These segments were termed proximal, middle and distal small intestine, respectively. The colon was isolated from the ileal-caecal junction to the anus, and the colonic tissue was taken from the middle of the segment. The tissues were collected in Lysing Matrix D homogenisation tube (MP Biomedicals) on dry ice and stored at − 80 °C before RNA extraction.

An additional cohort of C57BL6/JN mice fed 60% HFD was given metformin in the drinking water—the initial dose was 1 g/l for two weeks, which was, as the mice tolerated this well, increased to 3 g/l for an additional two weeks. Mice were sacrificed and intestinal tissue (cut into three equal sized small intestinal sections referred to as duodenum, jejunum and ileum, and two (proximal and distal) colon/rectum sections) were washed repeatedly in PBS to remove luminal contents and non-absorbed metformin. Other tissues taken included left liver lobe and left kidney and a terminal blood/plasma sample. All materials were stored at − 80 °C before mass spectroscopic quantification of metformin content.

### Analysis of metformin concentrations

Metformin was isolated utilising an adapted version of the liquid–liquid extraction previously described^69^. Briefly, the tissues were weighed (between ~ 2 to 20 mg) or biological fluids pipetted (~ 10 µL) into 2 mL screw cap Eppendorf plastic tube (Eppendorf, Stevenage, UK) along with a single 5 mm stainless steel ball bearing. Immediately after, 400 µL of chloroform:methanol (2:1, respectively) solution was added, followed by thorough mixing. Samples were then homogenised using a Bioprep 24-1004 homogeniser (Allsheng, Hangzhou City, China). 100 µL of the metformin-d6 internal standard, 1,1-Dimethyl-d6-biguanide hydrochloride from QMX laboratories (Thaxted, Essex, United Kingdom) (1 µM in water) was added followed by the addition of 600 µL of chloroform:methanol (2:1, respectively) solution and homogenised again. Then 300 µL of HPLC water was added to each sample. The samples were vortexed and centrifuged at ~ 21,000*g* for 5 min. The aqueous top layers were transferred into 2 mL amber glass vials and dried down using a Concentrator Plus system (Eppendorf, Stevenage, UK). Samples were reconstituted in 100 µL of the chromatography starting conditions (9:1 mix of mobile phase A and B) and thoroughly vortexed. Reconstituted samples were transferred into a 250 μL glass vial insert in a 2 mL amber glass vial ready for LC–MS analysis.

LC–MS analysis was achieved using a Shimadzu HPLC System (Shimadzu UK Limited, Milton Keynes, United Kingdom) with the injection of 5 µL of the sample onto a Scherzo SM-C18 column (150 mm × 3 mm I.D., 3 µm) maintained at 40 °C. Mobile phase A was 30 mM ammonium acetate in water with 0.02% acetic acid. Mobile phase B was 20% acetonitrile, 80% water with 0.8% acetic acid. The flow was maintained at 0.5 mL per minute with the following gradient: 0.00 min_10% mobile phase B; 0.20 min_10% mobile phase B; 1.20 min_99% mobile phase B; 5.00 min_99% mobile phase B; 5.10 min_10% mobile phase B; 8.00 min_10% mobile phase B. The sample injection needle was washed using 50:50 water and acetonitrile solution. The mass spectrometer used was the Thermo Scientific Exactive Orbitrap with a heated electrospray ionisation source (Thermo Fisher Scientific, Hemel Hempstead, UK). The mass spectrometer source tune file was optimised for metformin and applied to the instrument method. The metformin analysis was run in positive mode from 0 to 5.00 min with the mass spectrometer scan rate set at 2 Hz, giving a resolution set to 50,000 (arbitrary units) with a full-scan range of m/z 100 to 200.

### Statistical analysis

Results were analysed for normality distribution, and statistical differences between groups were analysed via GraphPad Prism 7.0 software. For RNA-seq data, statistical analysis of DE genes was performed via DE-Seq2 package using R Studio. The specific statistical tests used are stated in the figure legends. All data were considered statistically significant when P < 0.05.

## Supplementary Information

Supplementary Figures.

## Data Availability

Raw sequencing data from this study has been deposited in the GEO database with the accession number GSE165122.
