# Supplementary material for: Inhibition of mitochondrial function by metformin increases glucose uptake, glycolysis and GDF-15 release from intestinal cells
Source: Sci Rep. 2021 Jan 28;11:2529. doi: 10.1038/s41598-021-81349-7 (PMC7843649; doi:10.1038/s41598-021-81349-7)
Supplement: Supplementary file 1 — Supplementary Figures. [file 41598_2021_81349_MOESM1_ESM.pptx]

## Slide 1
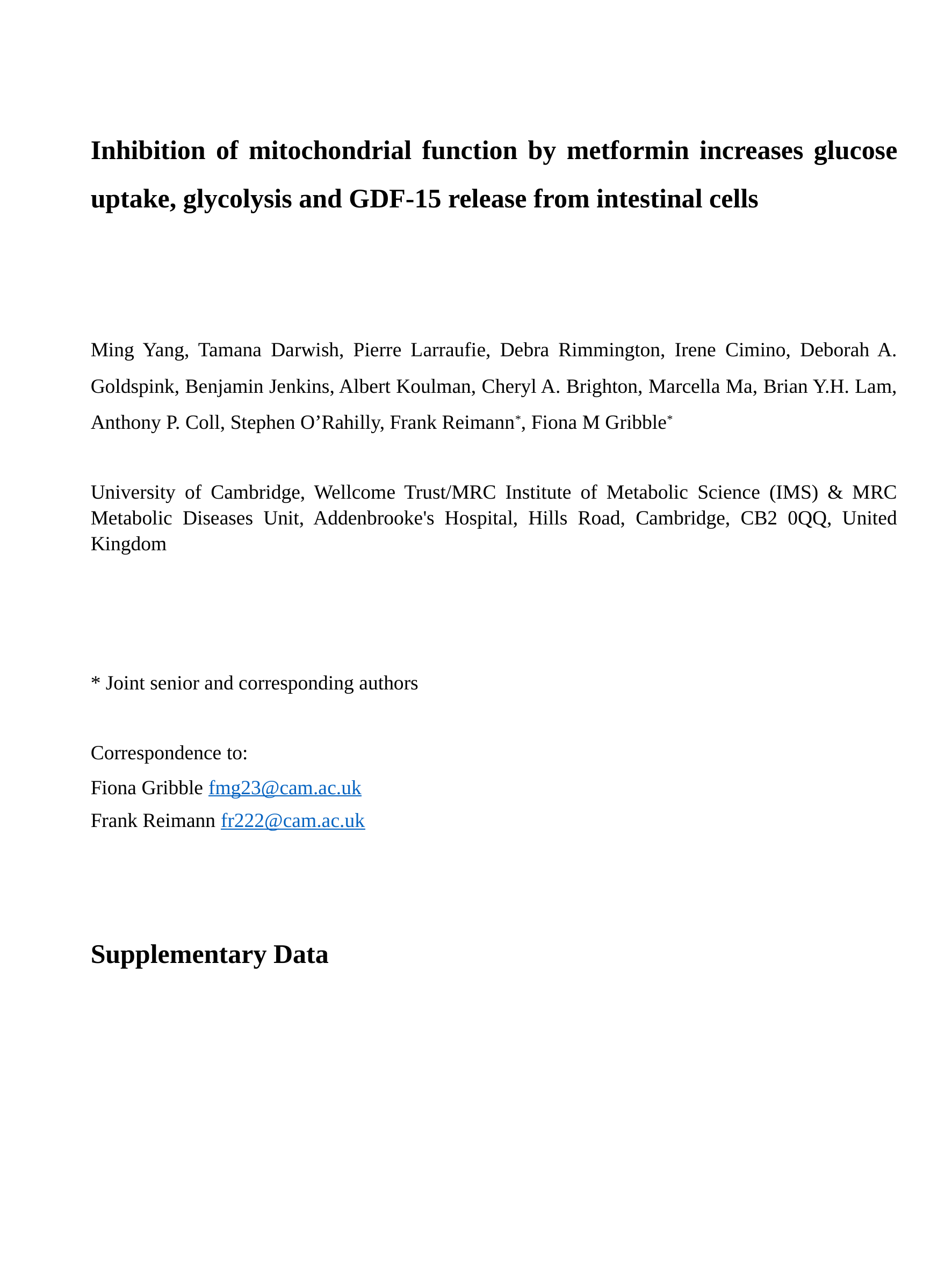

Inhibition of mitochondrial function by metformin increases glucose uptake, glycolysis and GDF-15 release from intestinal cells
Ming Yang, Tamana Darwish, Pierre Larraufie, Debra Rimmington, Irene Cimino, Deborah A. Goldspink, Benjamin Jenkins, Albert Koulman, Cheryl A. Brighton, Marcella Ma, Brian Y.H. Lam, Anthony P. Coll, Stephen O’Rahilly, Frank Reimann*, Fiona M Gribble*
University of Cambridge, Wellcome Trust/MRC Institute of Metabolic Science (IMS) & MRC Metabolic Diseases Unit, Addenbrooke's Hospital, Hills Road, Cambridge, CB2 0QQ, United Kingdom
* Joint senior and corresponding authors
Correspondence to:
Fiona Gribble fmg23@cam.ac.uk
Frank Reimann fr222@cam.ac.uk
Supplementary Data

## Slide 2
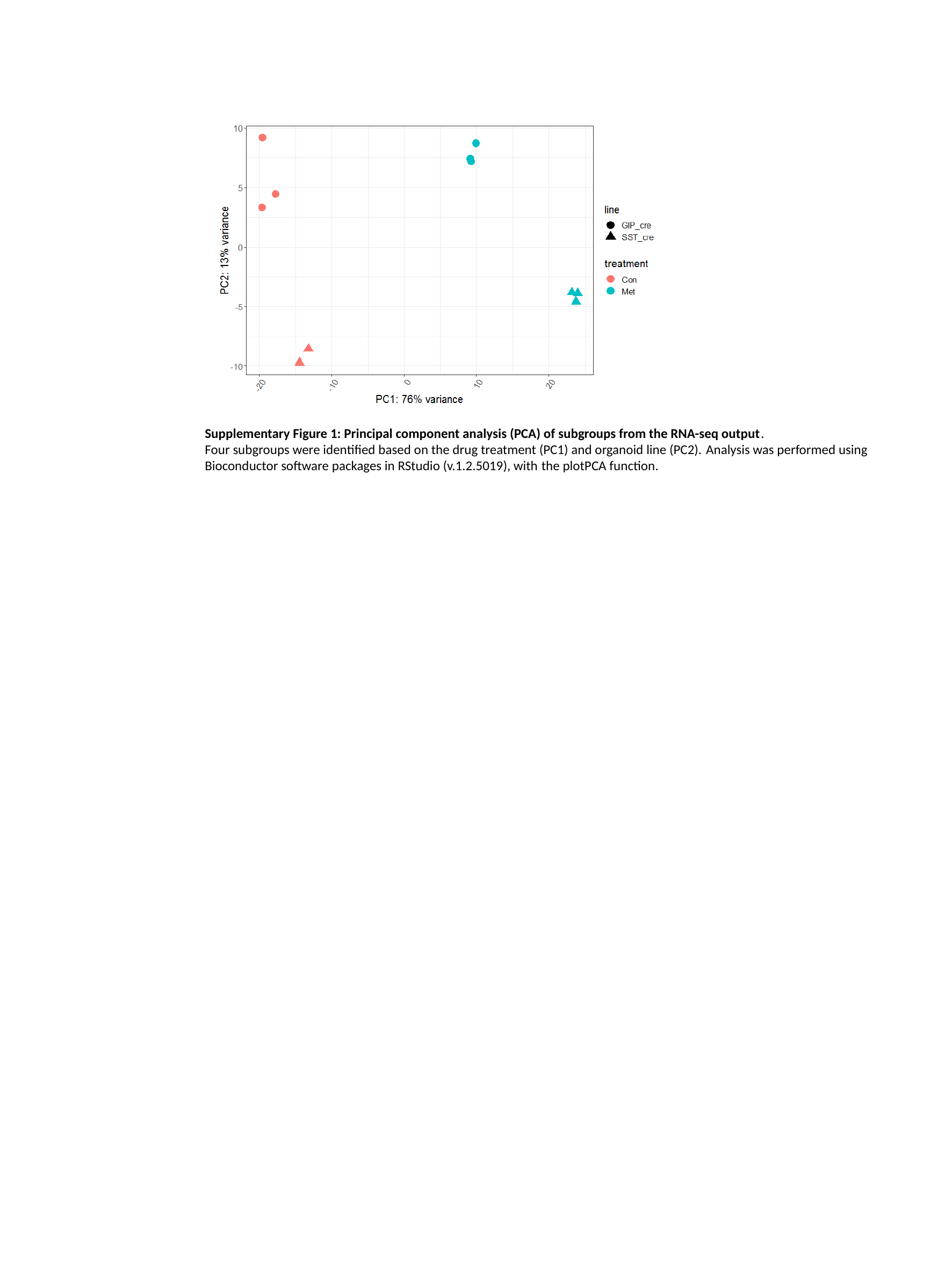

Supplementary Figure 1: Principal component analysis (PCA) of subgroups from the RNA-seq output.
Four subgroups were identified based on the drug treatment (PC1) and organoid line (PC2). Analysis was performed using Bioconductor software packages in RStudio (v.1.2.5019), with the plotPCA function.

## Slide 3
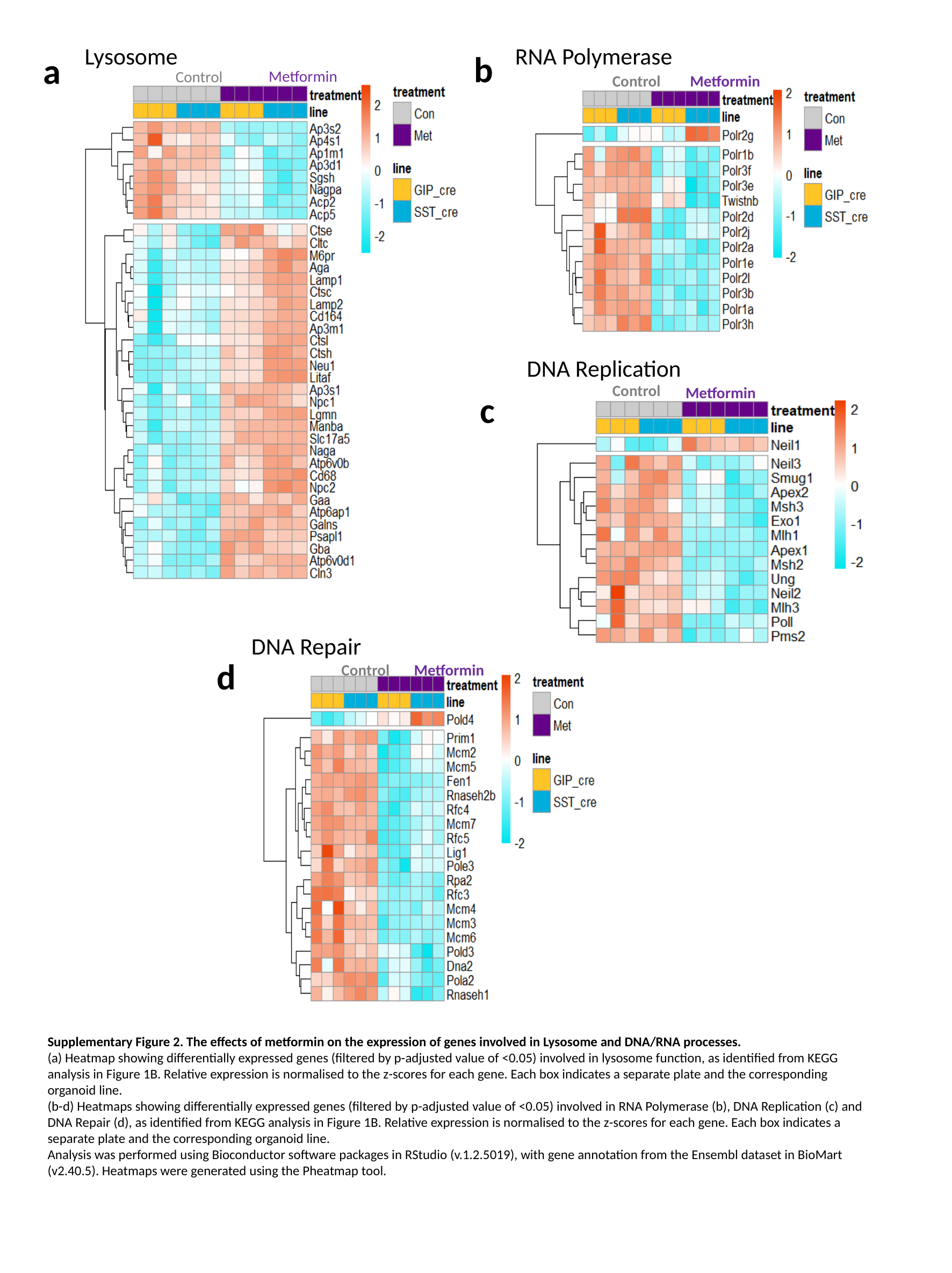

Lysosome
Metformin
Control
RNA Polymerase
b
a
Control
Metformin
DNA Replication
Control
Metformin
c
DNA Repair
d
Control
Metformin
Supplementary Figure 2. The effects of metformin on the expression of genes involved in Lysosome and DNA/RNA processes.
(a) Heatmap showing differentially expressed genes (filtered by p-adjusted value of <0.05) involved in lysosome function, as identified from KEGG analysis in Figure 1B. Relative expression is normalised to the z-scores for each gene. Each box indicates a separate plate and the corresponding organoid line.
(b-d) Heatmaps showing differentially expressed genes (filtered by p-adjusted value of <0.05) involved in RNA Polymerase (b), DNA Replication (c) and DNA Repair (d), as identified from KEGG analysis in Figure 1B. Relative expression is normalised to the z-scores for each gene. Each box indicates a separate plate and the corresponding organoid line.
Analysis was performed using Bioconductor software packages in RStudio (v.1.2.5019), with gene annotation from the Ensembl dataset in BioMart (v2.40.5). Heatmaps were generated using the Pheatmap tool.

## Slide 4
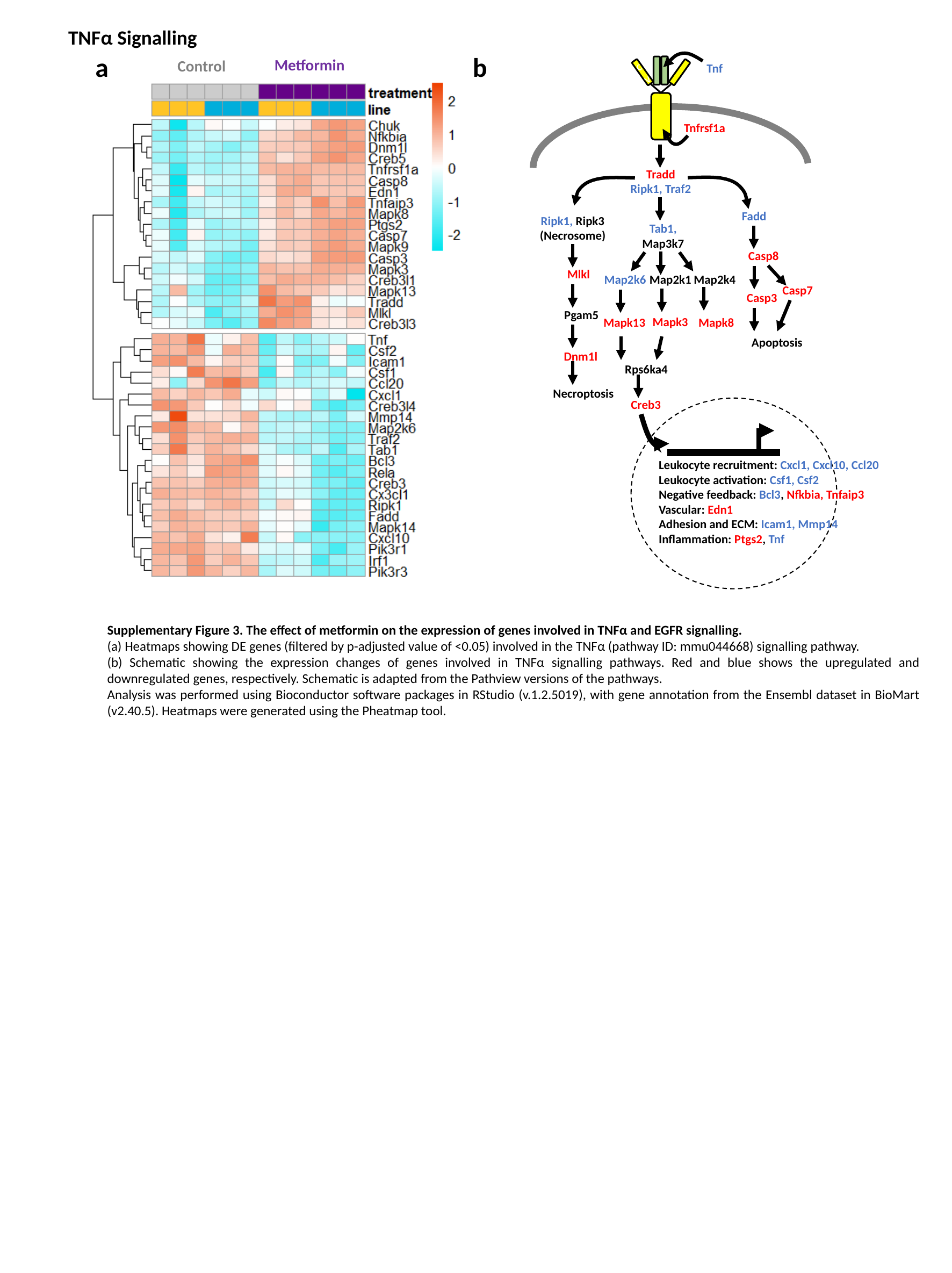

TNFα Signalling
a
b
Metformin
Control
Tnf
cv
Tnfrsf1a
Tradd
Ripk1, Traf2
Fadd
Ripk1, Ripk3
(Necrosome)
Tab1,
Map3k7
Casp8
Mlkl
Map2k6
Map2k1
Map2k4
Casp7
Casp3
Pgam5
Mapk3
Mapk8
Mapk13
Apoptosis
Dnm1l
Rps6ka4
Necroptosis
Creb3
Leukocyte recruitment: Cxcl1, Cxcl10, Ccl20
Leukocyte activation: Csf1, Csf2
Negative feedback: Bcl3, Nfkbia, Tnfaip3
Vascular: Edn1
Adhesion and ECM: Icam1, Mmp14
Inflammation: Ptgs2, Tnf
Supplementary Figure 3. The effect of metformin on the expression of genes involved in TNFα and EGFR signalling.
(a) Heatmaps showing DE genes (filtered by p-adjusted value of <0.05) involved in the TNFα (pathway ID: mmu044668) signalling pathway.
(b) Schematic showing the expression changes of genes involved in TNFα signalling pathways. Red and blue shows the upregulated and downregulated genes, respectively. Schematic is adapted from the Pathview versions of the pathways.
Analysis was performed using Bioconductor software packages in RStudio (v.1.2.5019), with gene annotation from the Ensembl dataset in BioMart (v2.40.5). Heatmaps were generated using the Pheatmap tool.

## Slide 5
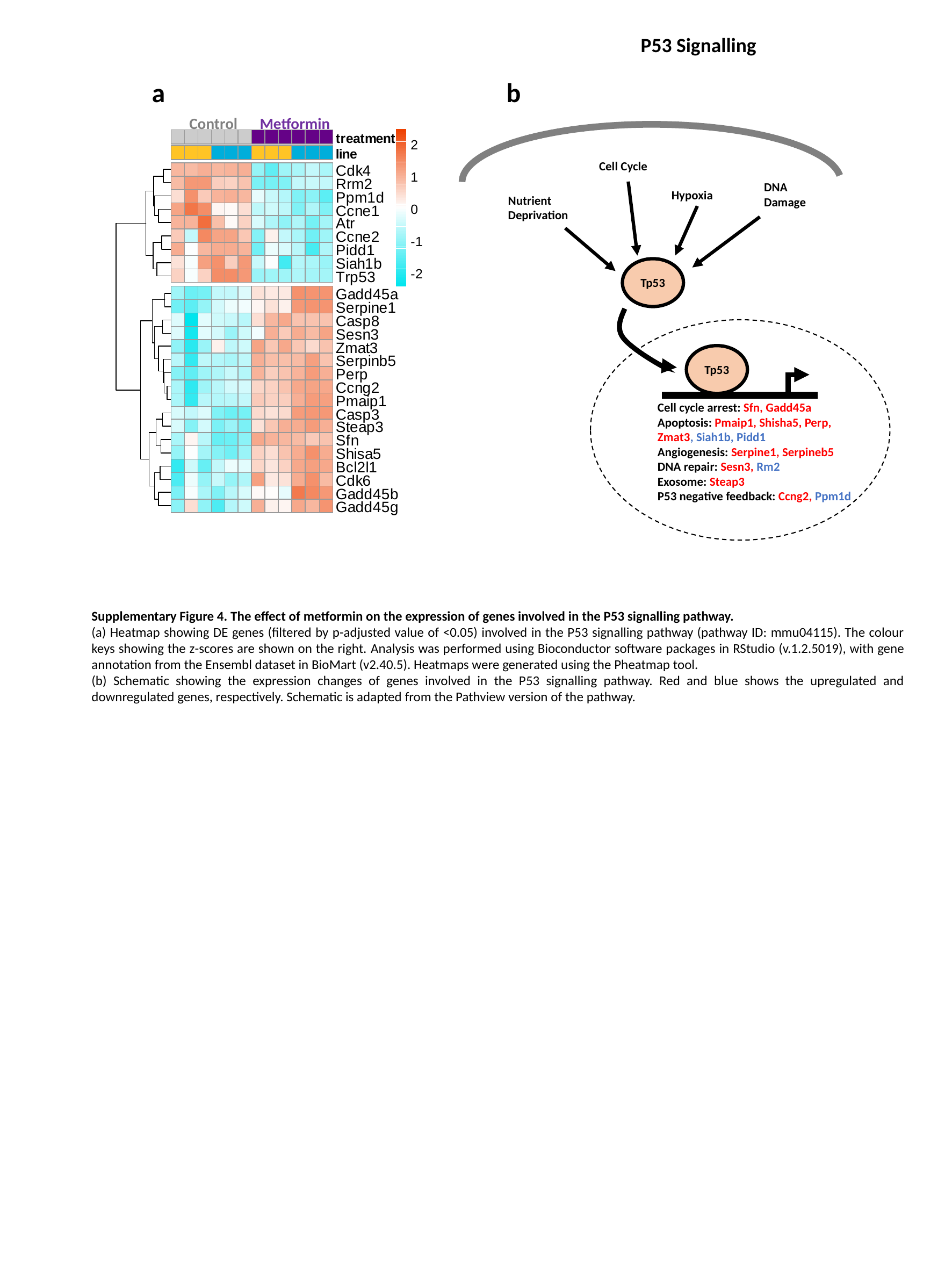

P53 Signalling
a
b
Control
Metformin
Cell Cycle
DNA
Damage
Hypoxia
Nutrient
Deprivation
Tp53
Tp53
Cell cycle arrest: Sfn, Gadd45a
Apoptosis: Pmaip1, Shisha5, Perp,
Zmat3, Siah1b, Pidd1
Angiogenesis: Serpine1, Serpineb5
DNA repair: Sesn3, Rm2
Exosome: Steap3
P53 negative feedback: Ccng2, Ppm1d
Supplementary Figure 4. The effect of metformin on the expression of genes involved in the P53 signalling pathway.
(a) Heatmap showing DE genes (filtered by p-adjusted value of <0.05) involved in the P53 signalling pathway (pathway ID: mmu04115). The colour keys showing the z-scores are shown on the right. Analysis was performed using Bioconductor software packages in RStudio (v.1.2.5019), with gene annotation from the Ensembl dataset in BioMart (v2.40.5). Heatmaps were generated using the Pheatmap tool.
(b) Schematic showing the expression changes of genes involved in the P53 signalling pathway. Red and blue shows the upregulated and downregulated genes, respectively. Schematic is adapted from the Pathview version of the pathway.

## Slide 6
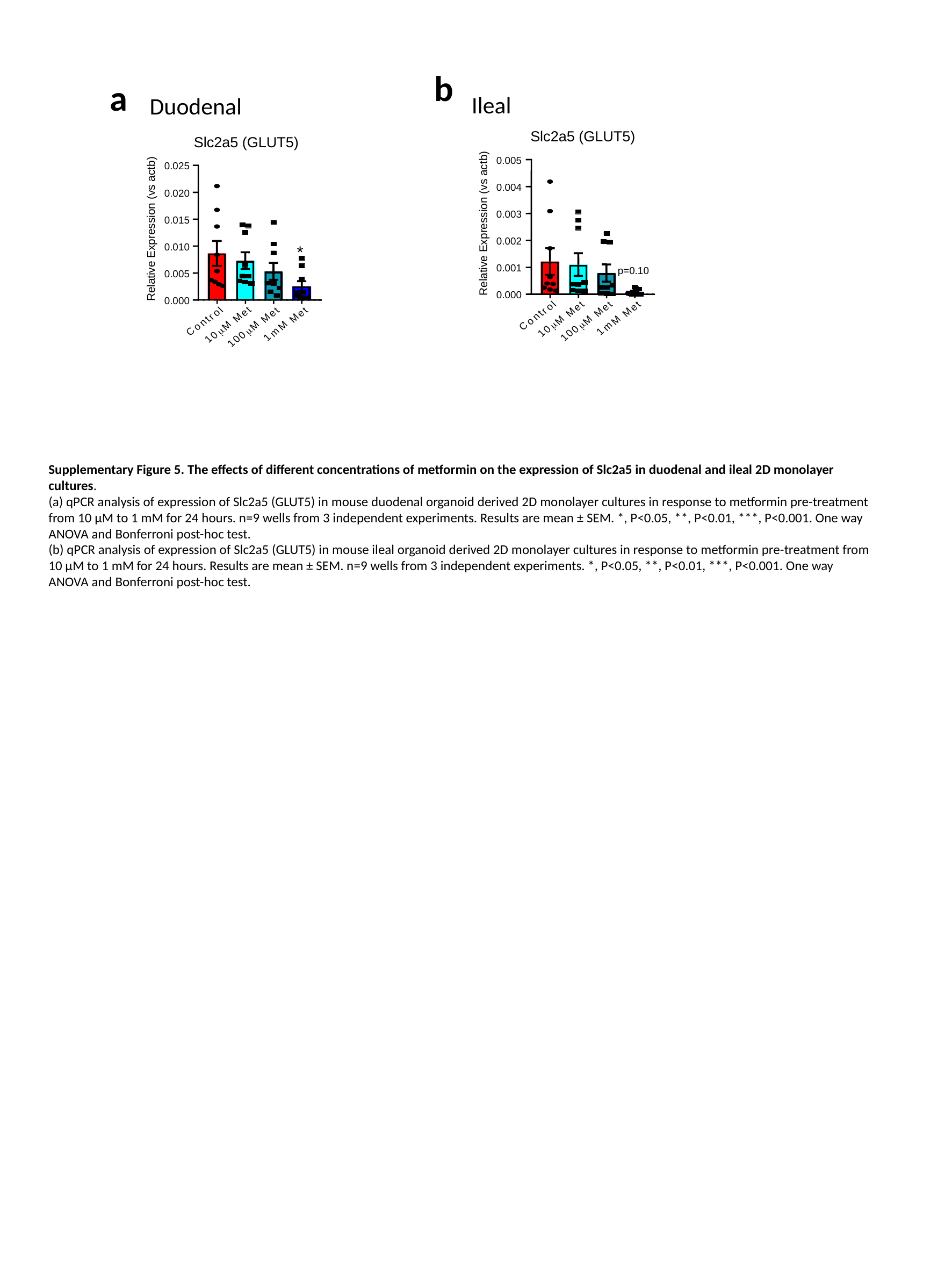

b
a
Ileal
Slc2a5 (GLUT5)
0.005
0.004
0.003
Relative Expression (vs actb)
0.002
0.001
p=0.10
0.000
l
t
t
t
o
e
e
e
r
M
M
M
t
n
M
M
M
o
m
m
C
m
0
0
1
1
0
1
Duodenal
Slc2a5 (GLUT5)
0.025
0.020
0.015
Relative Expression (vs actb)
0.010
*
0.005
0.000
l
t
t
t
o
e
e
e
r
M
M
M
t
n
M
M
M
o
m
m
C
m
0
0
1
1
0
1
Supplementary Figure 5. The effects of different concentrations of metformin on the expression of Slc2a5 in duodenal and ileal 2D monolayer cultures.
(a) qPCR analysis of expression of Slc2a5 (GLUT5) in mouse duodenal organoid derived 2D monolayer cultures in response to metformin pre-treatment from 10 µM to 1 mM for 24 hours. n=9 wells from 3 independent experiments. Results are mean ± SEM. *, P<0.05, **, P<0.01, ***, P<0.001. One way ANOVA and Bonferroni post-hoc test.
(b) qPCR analysis of expression of Slc2a5 (GLUT5) in mouse ileal organoid derived 2D monolayer cultures in response to metformin pre-treatment from 10 µM to 1 mM for 24 hours. Results are mean ± SEM. n=9 wells from 3 independent experiments. *, P<0.05, **, P<0.01, ***, P<0.001. One way ANOVA and Bonferroni post-hoc test.

## Slide 7
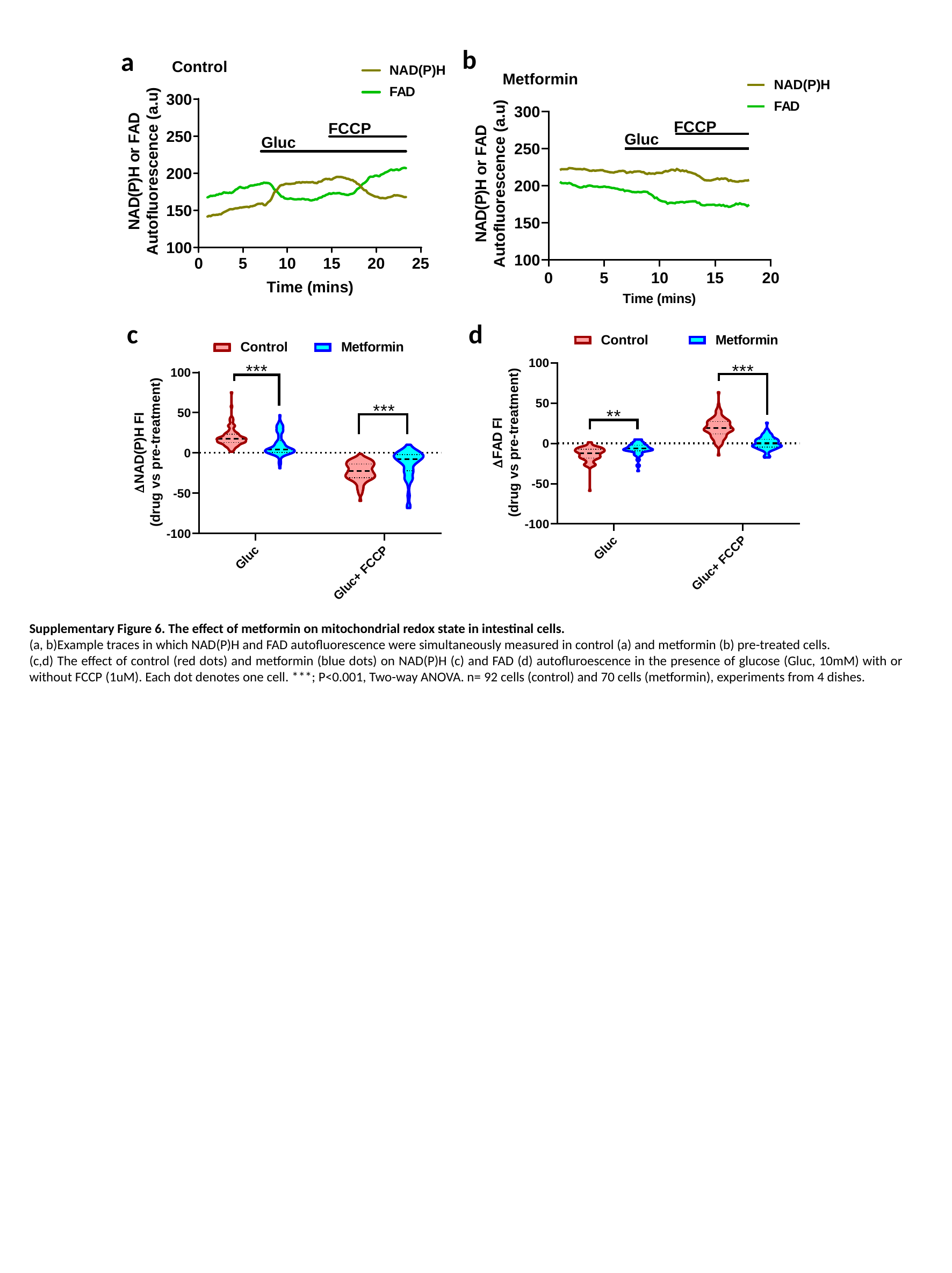

b
a
d
c
Supplementary Figure 6. The effect of metformin on mitochondrial redox state in intestinal cells.
(a, b)Example traces in which NAD(P)H and FAD autofluorescence were simultaneously measured in control (a) and metformin (b) pre-treated cells.
(c,d) The effect of control (red dots) and metformin (blue dots) on NAD(P)H (c) and FAD (d) autofluroescence in the presence of glucose (Gluc, 10mM) with or without FCCP (1uM). Each dot denotes one cell. ***; P<0.001, Two-way ANOVA. n= 92 cells (control) and 70 cells (metformin), experiments from 4 dishes.

## Slide 8
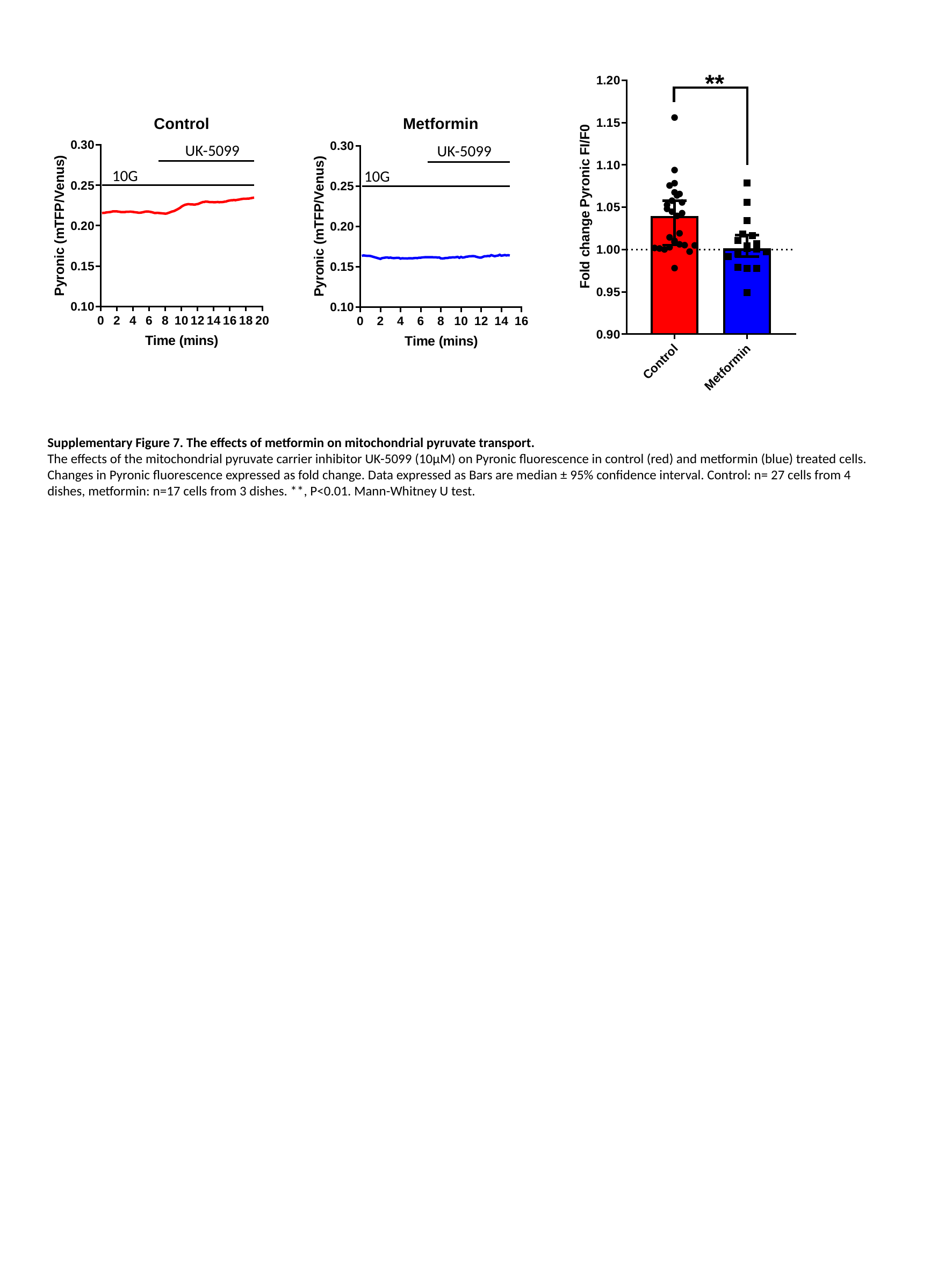

UK-5099
10G
UK-5099
10G
Supplementary Figure 7. The effects of metformin on mitochondrial pyruvate transport.
The effects of the mitochondrial pyruvate carrier inhibitor UK-5099 (10µM) on Pyronic fluorescence in control (red) and metformin (blue) treated cells. Changes in Pyronic fluorescence expressed as fold change. Data expressed as Bars are median ± 95% confidence interval. Control: n= 27 cells from 4 dishes, metformin: n=17 cells from 3 dishes. **, P<0.01. Mann-Whitney U test.

## Slide 9
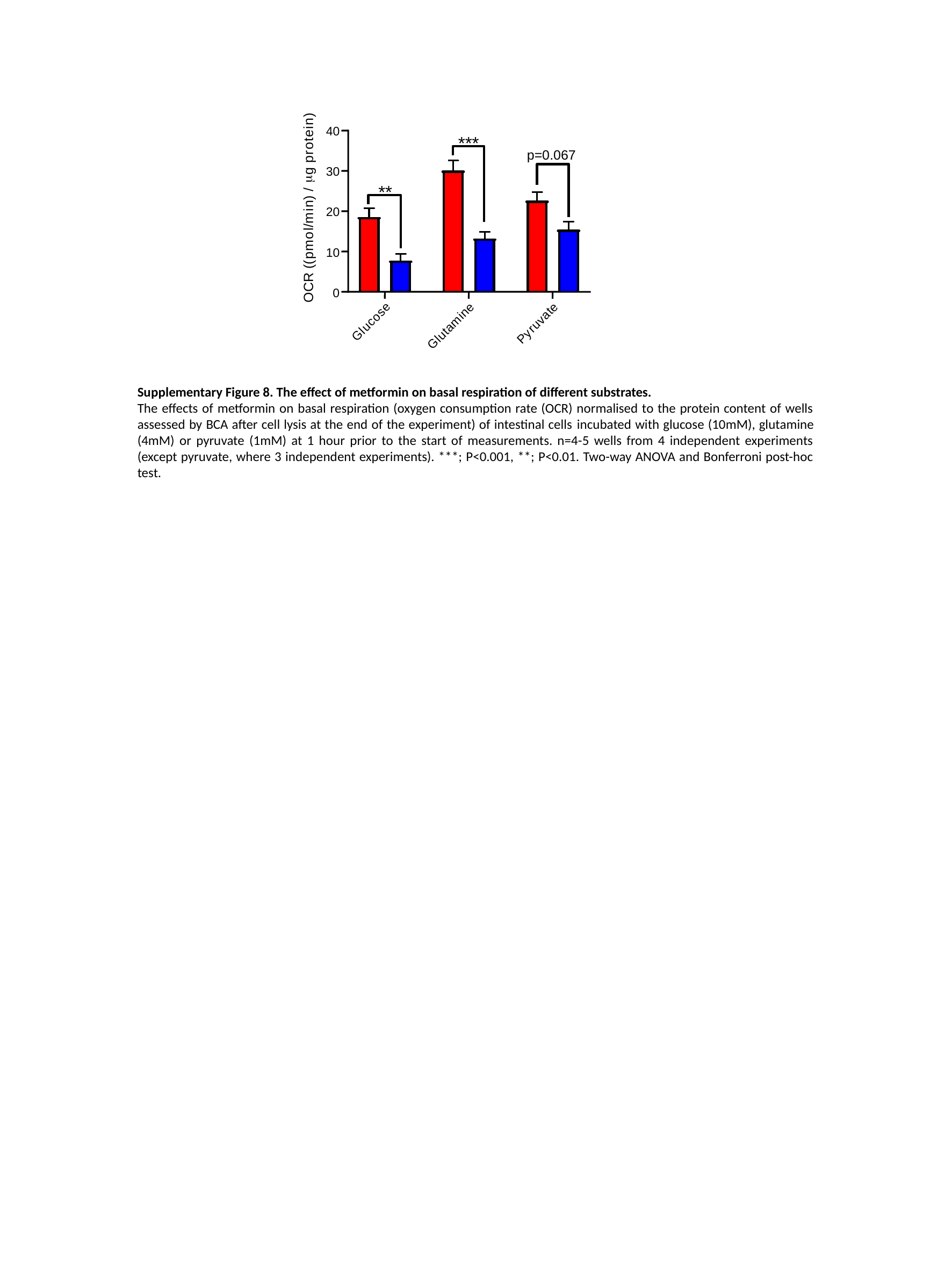

)
n
i
40
e
t
***
o
r
p=0.067
p
g
30
m
**
/
)
n
i
20
m
/
l
o
m
10
p
(
(
R
C
0
O
e
e
e
t
s
n
a
i
o
v
m
c
u
u
a
r
l
t
y
G
u
P
l
G
Supplementary Figure 8. The effect of metformin on basal respiration of different substrates.
The effects of metformin on basal respiration (oxygen consumption rate (OCR) normalised to the protein content of wells assessed by BCA after cell lysis at the end of the experiment) of intestinal cells incubated with glucose (10mM), glutamine (4mM) or pyruvate (1mM) at 1 hour prior to the start of measurements. n=4-5 wells from 4 independent experiments (except pyruvate, where 3 independent experiments). ***; P<0.001, **; P<0.01. Two-way ANOVA and Bonferroni post-hoc test.

## Slide 10
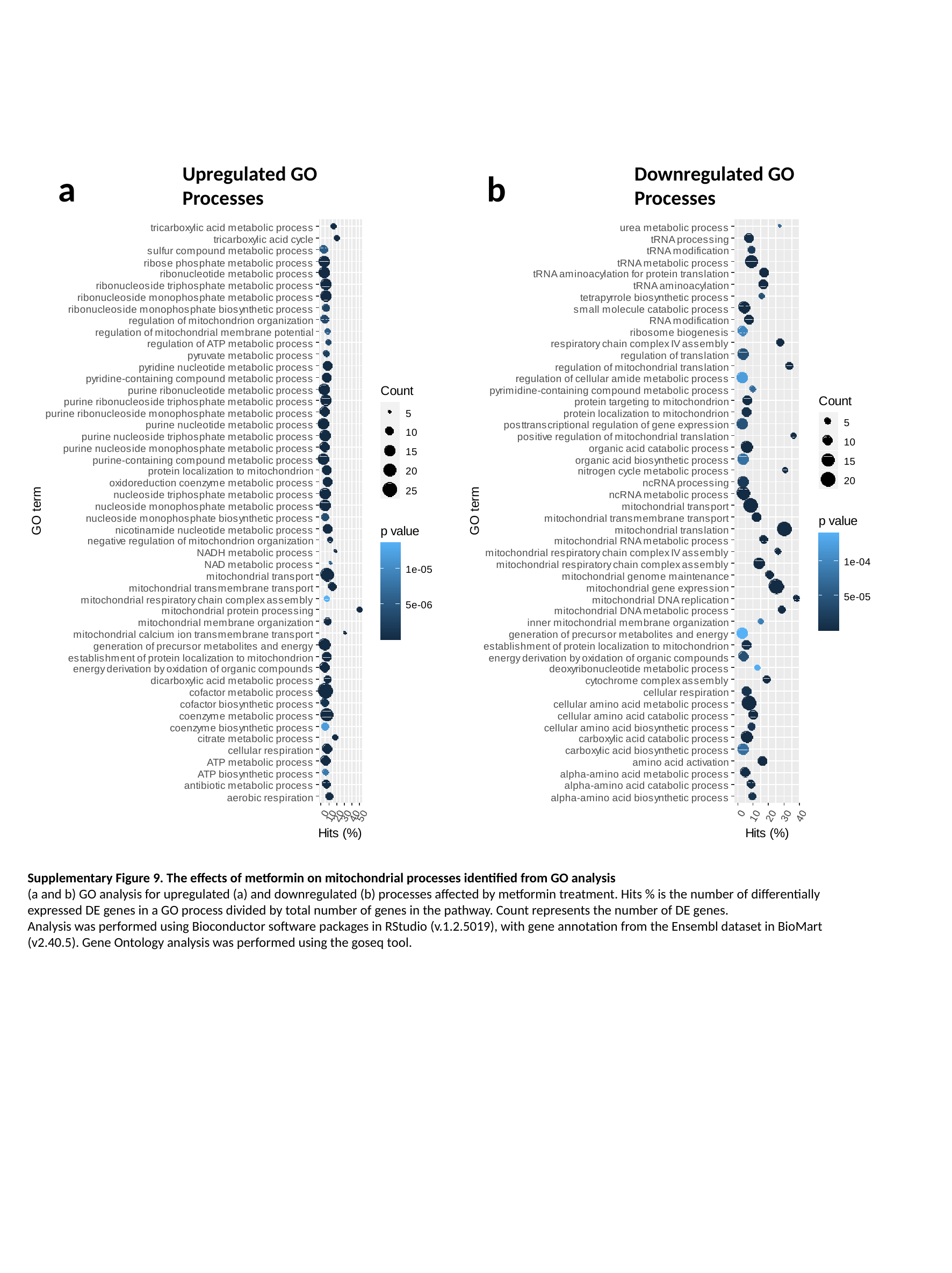

Upregulated GO
Processes
Downregulated GO
Processes
b
a
Supplementary Figure 9. The effects of metformin on mitochondrial processes identified from GO analysis
(a and b) GO analysis for upregulated (a) and downregulated (b) processes affected by metformin treatment. Hits % is the number of differentially expressed DE genes in a GO process divided by total number of genes in the pathway. Count represents the number of DE genes.
Analysis was performed using Bioconductor software packages in RStudio (v.1.2.5019), with gene annotation from the Ensembl dataset in BioMart (v2.40.5). Gene Ontology analysis was performed using the goseq tool.

## Slide 11
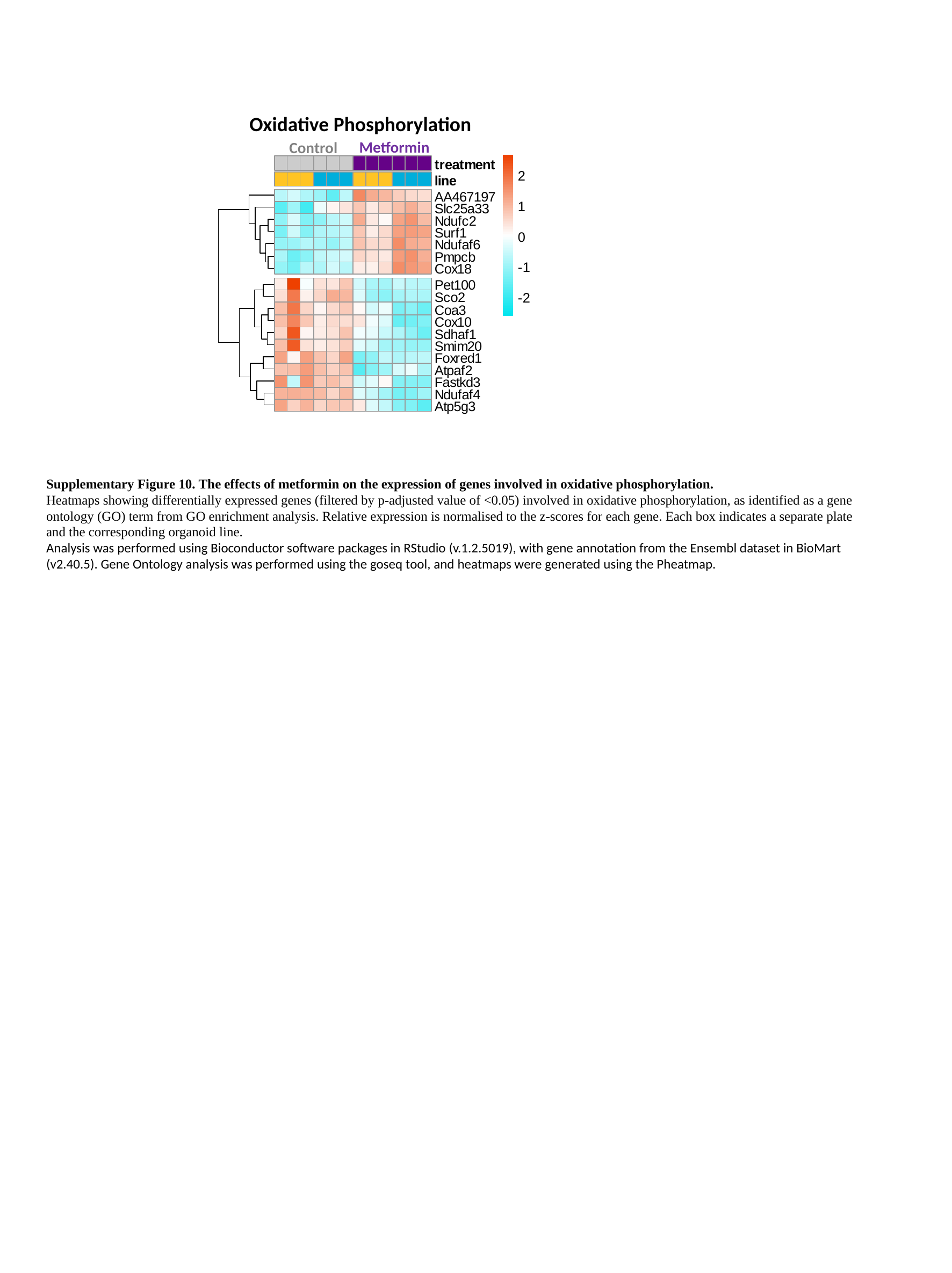

Oxidative Phosphorylation
Metformin
Control
Supplementary Figure 10. The effects of metformin on the expression of genes involved in oxidative phosphorylation.
Heatmaps showing differentially expressed genes (filtered by p-adjusted value of <0.05) involved in oxidative phosphorylation, as identified as a gene ontology (GO) term from GO enrichment analysis. Relative expression is normalised to the z-scores for each gene. Each box indicates a separate plate and the corresponding organoid line.
Analysis was performed using Bioconductor software packages in RStudio (v.1.2.5019), with gene annotation from the Ensembl dataset in BioMart (v2.40.5). Gene Ontology analysis was performed using the goseq tool, and heatmaps were generated using the Pheatmap.

## Slide 12
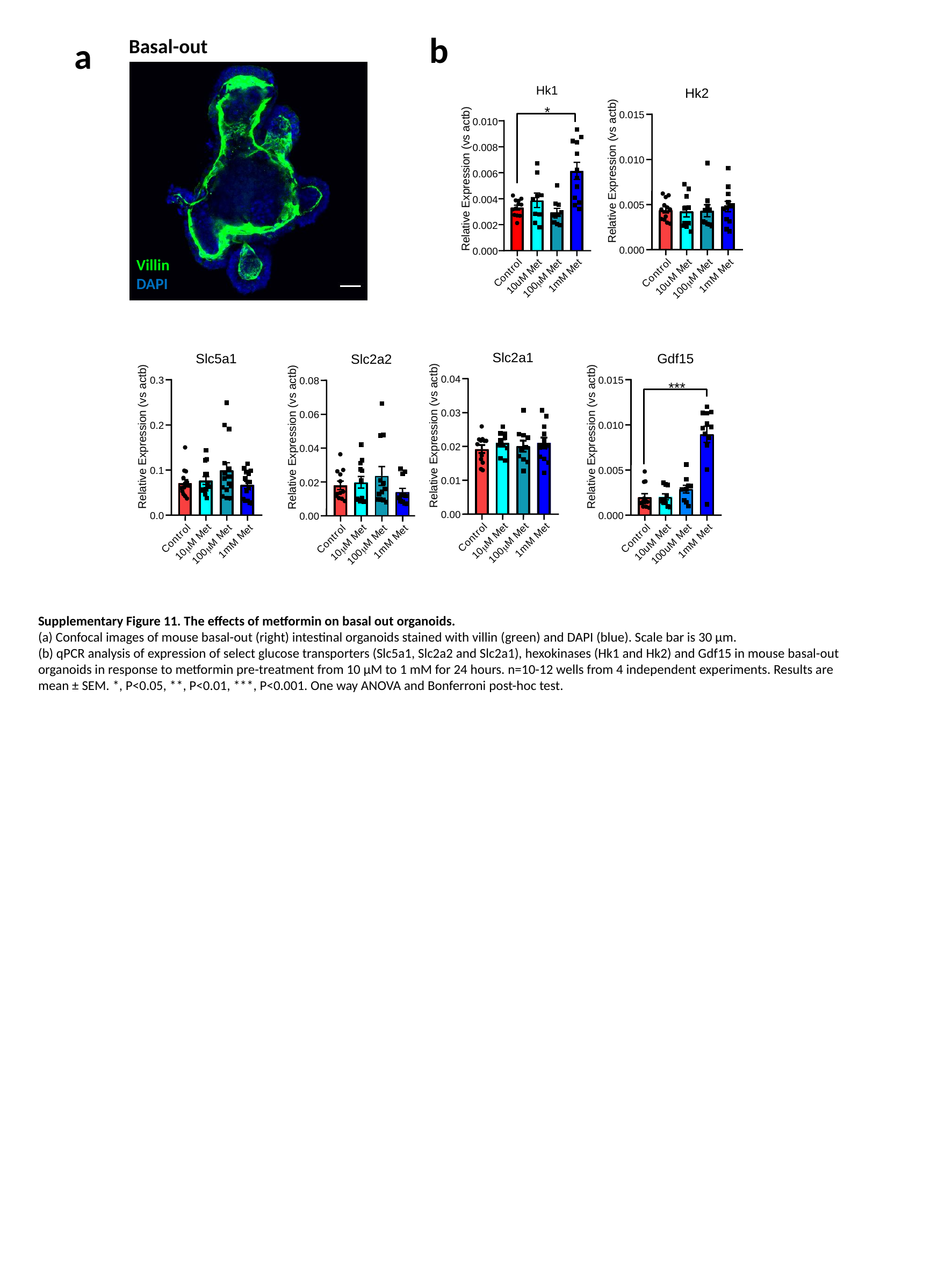

b
a
Basal-out
Hk1
*
0.010
0.008
0.006
Relative Expression (vs actb)
0.004
0.002
0.000
l
t
t
t
o
e
e
e
r
M
M
M
t
n
M
M
M
o
m
u
C
m
0
0
1
0
1
1
Hk2
0.015
0.010
Relative Expression (vs actb)
0.005
0.000
l
t
t
t
o
e
e
e
r
M
M
M
t
n
M
M
M
o
m
u
C
m
0
0
1
0
1
1
Villin
DAPI
Slc2a1
0.04
0.03
Relative Expression (vs actb)
0.02
0.01
0.00
l
t
t
t
o
e
e
e
r
M
M
M
t
n
M
M
M
o
m
m
C
m
0
0
1
1
0
1
Slc5a1
0.3
0.2
Relative Expression (vs actb)
0.1
0.0
l
t
t
t
o
e
e
e
r
M
M
M
t
n
M
M
M
o
m
m
C
m
0
0
1
1
0
1
Gdf15
0.015
***
0.010
Relative Expression (vs actb)
0.005
0.000
l
t
t
t
o
e
e
e
r
M
M
M
t
n
M
M
M
o
u
u
C
m
0
0
1
1
0
1
Slc2a2
0.08
0.06
Relative Expression (vs actb)
0.04
0.02
0.00
l
t
t
t
o
e
e
e
r
M
M
M
t
n
M
M
M
o
m
m
C
m
0
0
1
1
0
1
Supplementary Figure 11. The effects of metformin on basal out organoids.
(a) Confocal images of mouse basal-out (right) intestinal organoids stained with villin (green) and DAPI (blue). Scale bar is 30 µm.
(b) qPCR analysis of expression of select glucose transporters (Slc5a1, Slc2a2 and Slc2a1), hexokinases (Hk1 and Hk2) and Gdf15 in mouse basal-out organoids in response to metformin pre-treatment from 10 µM to 1 mM for 24 hours. n=10-12 wells from 4 independent experiments. Results are mean ± SEM. *, P<0.05, **, P<0.01, ***, P<0.001. One way ANOVA and Bonferroni post-hoc test.
